# Bioavailability, Efficacy, Safety, and Regulatory Status of Creatine and Related Compounds: A Critical Review

**DOI:** 10.3390/nu14051035

**Published:** 2022-02-28

**Authors:** Richard B. Kreider, Ralf Jäger, Martin Purpura

**Affiliations:** 1 Exercise & Sport Nutrition Lab, Human Clinical Research Facility, Department of Health & Kinesiology, Texas A&M University, College Station, TX 77843, USA; 2 Increnovo LLC, Milwaukee, WI 53202, USA; ralf.jaeger@increnovo.com (R.J.); martin.purpura@increnovo.com (M.P.)

**Keywords:** dietary ingredients, ergogenic aids, exercise, performance

## Abstract

In 2011, we published a paper providing an overview about the bioavailability, efficacy, and regulatory status of creatine monohydrate (CrM), as well as other “novel forms” of creatine that were being marketed at the time. This paper concluded that no other purported form of creatine had been shown to be a more effective source of creatine than CrM, and that CrM was recognized by international regulatory authorities as safe for use in dietary supplements. Moreover, that most purported “forms” of creatine that were being marketed at the time were either less bioavailable, less effective, more expensive, and/or not sufficiently studied in terms of safety and/or efficacy. We also provided examples of several “forms” of creatine that were being marketed that were not bioavailable sources of creatine or less effective than CrM in comparative effectiveness trials. We had hoped that this paper would encourage supplement manufacturers to use CrM in dietary supplements given the overwhelming efficacy and safety profile. Alternatively, encourage them to conduct research to show their purported “form” of creatine was a bioavailable, effective, and safe source of creatine before making unsubstantiated claims of greater efficacy and/or safety than CrM. Unfortunately, unsupported misrepresentations about the effectiveness and safety of various “forms” of creatine have continued. The purpose of this critical review is to: (1) provide an overview of the physiochemical properties, bioavailability, and safety of CrM; (2) describe the data needed to substantiate claims that a “novel form” of creatine is a bioavailable, effective, and safe source of creatine; (3) examine whether other marketed sources of creatine are more effective sources of creatine than CrM; (4) provide an update about the regulatory status of CrM and other purported sources of creatine sold as dietary supplements; and (5) provide guidance regarding the type of research needed to validate that a purported “new form” of creatine is a bioavailable, effective and safe source of creatine for dietary supplements. Based on this analysis, we categorized forms of creatine that are being sold as dietary supplements as either having strong, some, or no evidence of bioavailability and safety. As will be seen, CrM continues to be the only source of creatine that has substantial evidence to support bioavailability, efficacy, and safety. Additionally, CrM is the source of creatine recommended explicitly by professional societies and organizations and approved for use in global markets as a dietary ingredient or food additive.

## 1. Introduction

Creatine (N-(aminoiminomethyl)-N-methyl glycine) is a naturally occurring nitrogen-containing compound that plays an integral role in cellular metabolism. While creatine is commonly referred to as an amino acid, it is not actually an amino acid in the traditional sense. It is not incorporated into proteins or an essential, conditionally essential, and non-essential amino acid that serves as building blocks of protein. Instead, creatine is an amino acid derivative that is endogenously synthesized from the amino acids arginine and glycine by L-arginine: glycine amidinotransferase (AGAT) to guanidinoacetate (GAA). The GAA is then methylated (i.e., CH_3_ group added) by the enzyme guanidinoacetate N-methyltransferase (GAMT) with S-adenosyl methionine (SAMe) to form creatine [1]. The kidney, liver, pancreas, and some areas within the brain contain AGAT. Most GAA is formed in the kidney and converted by GMAT to creatine in the liver [2,3,4]. The primary role of creatine is to bind with inorganic phosphate (Pi) in the cell to form phosphocreatine (PCr), and thereby serve as a high-energy phosphate source of energy to resynthesize adenosine triphosphate (ATP) that has been degraded to adenosine diphosphate (ADP) + Pi as a source of energy to fuel cellular metabolism [5]. Creatine also plays a critical role in translocating energy-related intermediates from the electron transport system in the mitochondria to the cytosol [6,7].

About 95% of creatine is stored in the muscle, with the remaining amount found in other tissues like the heart, brain, and testes [8,9]. About two-thirds of creatine is bound with Pi and stored as PCr with the remaining one-third stored as free creatine (Cr). The total creatine pool (Cr + PCr) is about 120 mmol/kg of dry muscle mass for an individual who consumes a diet with red meat and fish [10]. The body breaks down about 1–2% of the intramuscular creatine pool into creatinine, which is excreted in the urine [10,11,12]. Daily degradation of creatine to creatinine is greater in individuals with larger muscle mass and individuals with higher levels of physical activity [13]. Creatine synthesis provides about half of the daily need for creatine [2]. The remaining creatine needed to maintain normal tissue levels is obtained in the diet primarily from red meat and fish [14,15,16,17] or dietary supplements containing a bioavailable source of creatine [14,15,16]. Since creatine stores are not fully saturated on vegan or omnivorous diets that typically provide 0 to 1.5 g/day of creatine, daily dietary creatine needs have been estimated to be 2–4 g/day [2,6,15]. For this reason, dietary supplementation of creatine has been recommended to optimize creatine stores [5,6]. The most extensively studied and effective form of creatine found in nutritional supplements that professional organizations recommend for use is creatine monohydrate (CrM) [14,15,17,18]. Over the last 30 years, several studies have shown that CrM supplementation (e.g., 5 g/day for 5–7 days or 3–5 g/day for 30 days) increases blood, muscle, and tissue levels of creatine and PCr by 20–40% [11,12,19,20]. Co-ingesting CrM with carbohydrates [20,21,22] and carbohydrate and protein [23] promotes more consistent and greater creatine retention. The increased creatine levels have been reported to enhance high-intensity exercise performance and exercise training adaptations [14,15,24]. Furthermore, there is accumulating research that CrM supplementation may have health and clinical benefits in populations that may benefit from increasing creatine availability to the cell [5].

In 2011, we published a paper overviewing the known pharmacokinetics, bioavailability, efficacy, and regulatory status of various purported “forms” of creatine “marketed with claims of improved physical, chemical, and physiological properties in comparison to CrM” [25]. The article concluded that “the efficacy, safety, and regulatory status of most of the newer forms of creatine found in dietary supplements have not been well-established” and “there is little to no evidence supporting marketing claims that these newer forms of creatine are more stable, digested faster, more effective in increasing muscle creatine levels, and/or associated with fewer side effects than CrM” [25]. Similarly, in its most recent position stand on creatine supplementation, the International Society of Sport Nutrition (ISSN) concluded: (1) “Creatine monohydrate is the most extensively studied and clinically effective form of creatine for use in nutritional supplements in terms of muscle uptake and ability to increase high-intensity exercise capacity”; (2) “Claims that different forms of creatine are degraded to a lesser degree than creatine monohydrate in vivo or result in a greater uptake to muscle are currently unfounded [25]”; and, (3) “Clinical evidence has not demonstrated that different forms of creatine such as creatine citrate [22], creatine serum [26], creatine ethyl ester [27], buffered forms of creatine [28], or creatine nitrate [29] promote greater creatine retention than creatine monohydrate [25].” These conclusions were reiterated more recently in a review related to myths and misconceptions about creatine supplementation noting “there are no peer-reviewed published papers showing that the ingestion of equal amounts of creatine salts [30,31,32,33] or other forms of creatine like effervescent creatine [22], creatine ethyl ester [27,34,35], buffered creatine [28], creatine nitrate [29,36], creatine dipeptides, or the micro amounts of creatine contained in creatine serum [26,37,38] and beverages (e.g., 25–50 mg) increases creatine storage in muscle to a greater degree than creatine monohydrate [25]. Most studies show that ingestion of these other forms have less physiological impact than creatine monohydrate on intramuscular creatine stores and/or performance and that any performance differences were more related to other nutrients that creatine is bound to or co-ingested within supplement formulations.”

Despite this clear scientific evidence, guidance from professional organizations, and regulatory challenges of selling other forms of creatine in the global marketplace, companies continue to market “new forms” of creatine that are purportedly more stable, bioavailable, and/or effective than CrM. Some have argued that mentioning some of the forms of creatine described in our 2011 paper served as validation or an endorsement that these forms of creatine were valid sources of creatine rather than the intended conclusion that these purported forms of creatine were not scientifically validated, safe, effective, and regulatory approved sources of creatine for dietary supplements. The intent of this comprehensive review is to provide an update regarding (1) how creatine is absorbed from food and/or dietary supplements into the body; (2) whether sources of creatine currently marketed and/or used in dietary supplements are bioavailable sources of creatine; and (3) whether any of these purportedly alternate forms of creatine are as effective in increasing creatine stores in the body to a greater degree than CrM. Based on this assessment, compounds commonly marketed as sources of creatine will be categorized as: (1) Strong evidence to support bioavailability, efficacy, and safety; (2) limited evidence to support bioavailability, efficacy, and safety; or (3) no evidence to support bioavailability, efficacy, and safety.

## 2. Methods

**Figure 1:**
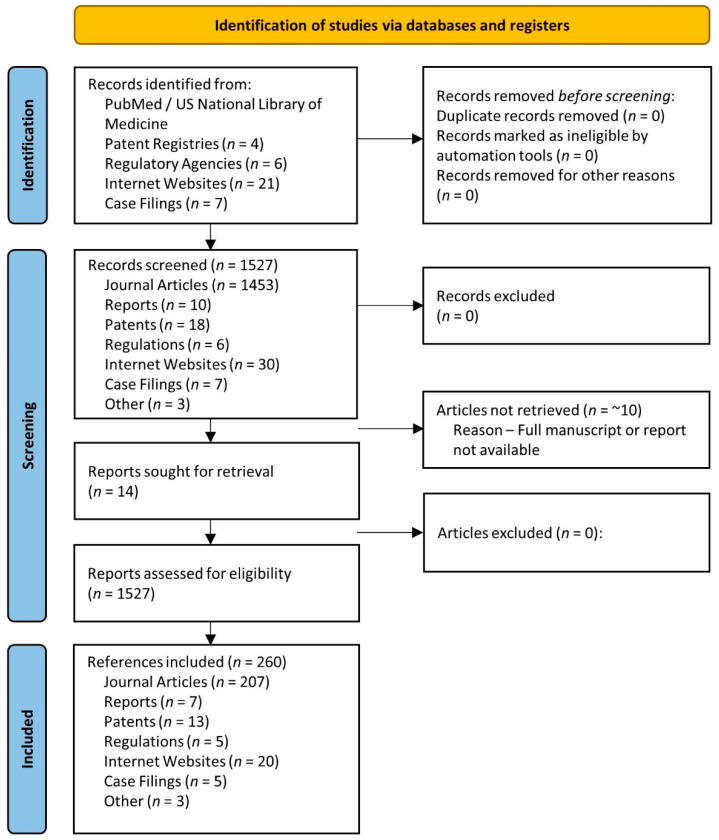
PRISMA flow chart.

This paper was conducted as a systematic review of the literature related to sources of creatine marketed as ingredients for dietary supplements or found in dietary supplements, food products, and/or beverages marketed as containing creatine. This was accomplished by performing a PubMed search of the US National Library of Medicine database of different sources of creatine found in dietary supplements related to solubility, stability, bioavailability, supplementation, and performance. In addition, we conducted patent searches of the United States Patent and Trademark Office, the European Patent Office, the Japan Patent Office, and World Intellectual Property Organization (WIPO) mainly when no articles were found from a PubMed search on the purported form of creatine. We also reviewed company websites to assess claims and studies they cited to support claims, as well as publicly available reports of studies submitted as evidence to the court in cases related to these purported forms of creatine. To assess the current regulatory status, we conducted a search of key markets around the world, including the US Food and Drug Administration (FDA); the Therapeutic Goods Administration in the Department of Health of Australia; Health Canada; the State Administration for Market Regulation of China; the European Union Commission; the Japan Ministry of Health, Labor and Welfare; and the Korea Food and Drug Administration. The information obtained from this search was used to assess the legal and regulatory status related to the sale of purported forms of creatine in these marketplaces. Figure 1 shows a Preferred Reporting Items for Systematic Reviews and Meta-Analyses (PRISMA) flow chart [39].

## 3. Bioavailability

Bioavailability refers to the degree or rate at which a drug or substance is absorbed into the body, reaches the intended target site, and is available to influence physiological activity [40]. In terms of nutrients, bioavailability refers to the amount of the nutrient contained in the food or supplement that is delivered to the target tissue and available in the intended tissue for metabolic activity [40]. If food or supplement contains a large number of nutrients, but only a small percentage of the nutrient is liberated from the food or supplement, transported through the blood to the tissue, and ultimately taken up by the tissue, it is not very bioavailable. Similarly, if a similar quantity of food or supplement delivers less of the active nutrient to the target tissue than another food or supplement of equal quantity, it is comparatively less bioavailable. In the case of creatine, individuals who consume meat and fish in their diet typically have a plasma creatine level of around 25 µmol/L (about 3.75 µg/mL), and a muscle creatine content of about 120 mmol/kg dry muscle mass (DMM) [11,12,19,20]. Muscle creatine levels are typically lower in individuals following a vegan diet [41,42] and the elderly, who may not consume as much protein in their diet or have more difficulty digesting dietary protein [43,44]. Plasma creatine levels increase after consuming creatine-containing food and dietary supplements containing a bioavailable source of creatine in proportion to the amount creatine ingested and digestion rate [2,41,42,45,46].

For a dietary source of creatine to be bioavailable, creatine must be: (1) absorbed as creatine into the blood and transported to tissues [2,25]; (2) transported into tissue via tissue-specific creatine transporter genes (e.g., CRT1 or SLC6A8 in muscle) [6,7]; and (3) increase tissue and cellular creatine and PCr content by a physiologically meaningful amount to influence metabolic activity (e.g., 20–40% in muscle and 10–20% in brain) [5,12,15,20,24,47]. In creatine research, the efficacy of creatine supplementation is determined by assessing the magnitude in which creatine supplementation protocol increases muscle creatine content as typically measured from muscle biopsy samples and/or muscle and brain creatine content as determined from magnetic resonance spectroscopy (MRS) [15]. Since oral ingestion of CrM is nearly 100% bioavailable (i.e., it’s either absorbed by tissue or excreted in urine), whole-body creatine retention with CrM supplementation can also be estimated as the difference between daily intake of CrM and urinary creatine output [22]. Purported forms of creatine that do not increase blood creatine concentrations, do not increase uptake of creatine through tissue-specific creatine transporters, and ultimately do not increase tissue creatine levels by physiologically meaningful amounts would not affect creatine-related metabolic function. This is regardless of whether the purported form of creatine is more solubility in water, is more stable under various temperature and pH conditions outside of the body, or is delivered in food, gel, and liquid sources, as will be discussed below.

**Figure 2:**
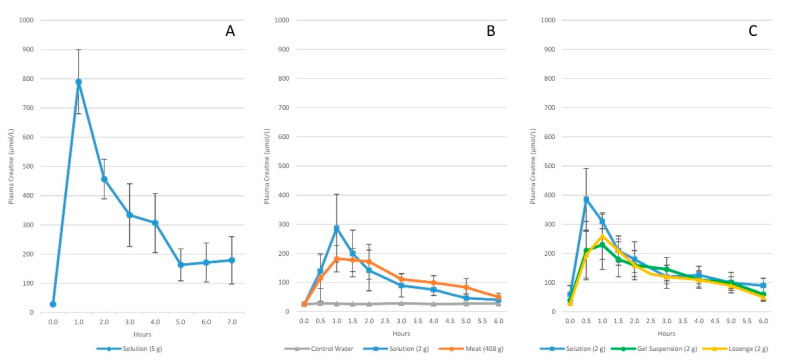
Changes in plasma creatine concentrations after administration of 5 g of creatine monohydrate (CrM) in solution (**A**) [12]; water, 2 g of CrM administered in solution, or 408 g of slightly cooked meat containing 5.4 g of creatine (**B**) [45]; or 2 g of CrM provided in solution, gel suspension, or in a hard candy lozenge (**C**) [45].

In support of this contention, classic experiments by Harris et al. [12] indicated that a dose of orally ingested creatine should ideally increase plasma creatine levels to greater than 500 µmol/L (75 µg/mL) to optimize tissue uptake. They reported that oral ingestion of 1 g or less of CrM had negligible effects on blood creatine content (i.e., rarely exceeding 100 µmol/L (15 µg/mL)). However, ingestion of one oral dose of 5 g of CrM (equivalent to about 1.1 kg of uncooked beef) resulted in plasma creatine levels of about 800 µmol/L (120 µg/mL) after 1 h of ingestion. It sustained plasma creatine above 200 µmol/L (30 µg/mL) for over 4–5 h (see Figure 2A). Supplementation of 5 g of CrM every 2 h maintained peak plasma creatine to levels exceeding 1000 µmol/L (150 µg/mL). Moreover, ingesting 5 g of CrM, 4 to 6 times a daily for 2-days or more significantly increased muscle creatine content of about 35%. Creatine uptake into the muscle was greatest during the first two days of CrM supplementation and declined over the next few days as muscle creatine levels became fully saturated. Subsequent studies from Hultman and colleagues [11] investigated the effects of various CrM supplementation strategies on changes in muscle creatine content. These experiments revealed that: (1) consuming 4 × 5 g of CrM per day for 6-days (i.e., creatine loading strategy) significantly increased muscle free creatine content by 33% and returned to baseline within 4-weeks after supplementation; (2) ingesting 4 × 5-g doses of CrM for 6-days followed by ingestion of 2 g/day of CrM for 28 days maintained a 36% increase in muscle creatine levels; and (3) ingesting 3 g/day of CrM for 35 days (i.e., low dose supplementation strategy) resulted in a gradual 16.7% increase in muscle creatine content. About 70% of the increase in the total creatine pool was observed in changes in free creatine content in the muscle.

Harris and associates [45] also conducted two experiments assessing the bioavailability of CrM in solution compared to creatine obtained from meat, crushed lozenges, and suspended in gel (see Figure 2B,C). In the first study, the researchers reported that ingestion of 2.5 g of CrM in solution (providing about 2.2 g of creatine) promoted a more rapid and greater increase in peak plasma creatine (287 ± 115 µmol/L or 42 µg/mL) than ingesting 408 g of lightly cooked steak containing 5.4 g (182 ± 52 µmol/L or 27 µg/mL). However, ingesting 5.4 g of creatine in lightly cooked meat promoted a more sustained increase in plasma creatine. Nevertheless, both strategies resulted in similar increases in area under the curve (AUC) values (507 ± 205 and 518 ± 153 µmol/h/L, respectively). In the second study, the researchers reported that ingestion of 2 g of CrM in solution resulted in peak plasma creatine levels of 386 ± 88 µmol/L (57 µg/mL) with an AUC of 622 ± 193 µmol/h/L. This compared to the peak value of 269 ± 67 µmol/L (41 µg/mL) with an AUC of 399 ± 196 µmol/h/L when CrM was administered in suspended gel and a peak creatine level of 277 ± 53 µmol/L and an AUC of 438 ± 131 µmol/h/L when CrM was administered as crushed lozenges. Collectively, these findings and are important because they demonstrate: (1) ingestion of 5 g of creatine from meat or 2–2.5 g of CrM administered in fluid, gels, and solids increased plasma creatine content by physiologically significant amounts needed to promote creatine uptake into the muscle; (2) the optimal single dose of CrM to increase plasma creatine levels is 5 g, but that ingesting 2–3 g will increase plasma creatine to sufficient levels to promote creatine uptake to tissue; (3) ingesting 5 g of CrM, 4 to 6 times a day for as little as 2 days was sufficient to significantly increase muscle creatine levels; (4) ingesting 5 g of CrM, 4 times a day for 6 days (i.e., 120 g total) increased muscle free creatine by about 35%; (5) consuming 3 g/day of CrM for 35 days (i.e., 105 g total) promoted a gradual 16.7% increase in muscle creatine; and (6) they provided a scientific basis for CrM supplementation recommendations and data for comparison of the efficacy of other purported forms of creatine marketed in dietary supplements [15].

### 3.1. Methods to Assess Bioavailability

#### 3.1.1. Assess Chemical Structure

The first step in assessing the bioavailability of a purported novel form of creatine is to determine whether the purported source of creatine contains a creatine molecule. Although this seems obvious, as will be seen below, some purported sources of creatine do not contain the complete creatine molecule. Instead, they only contain a portion of the creatine molecule structure or rearrange the chemical structure such that the compound is not really creatine. Moreover, some purported sources of creatine bind or complex compounds to creatine (or a portion of its structure) with bonds so strong (e.g., amide bonds) they would not likely break down into creatine through normal digestion, and therefore would not likely increase creatine levels in the blood or tissues. In other words, they are simply not bioavailable sources of creatine. Consequently, assessing the chemical structure of a purported form of creatine and whether associated bonds would easily disassociate into a creatine molecule is the first question that should be asked when evaluating claims about a purported “novel form” of creatine.

#### 3.1.2. Assess Changes in Blood Creatine Content

The next step is to determine if the purported source of creatine is absorbed through normal digestive processes into the blood and increases plasma creatine levels in a physiologically significant manner. Purported forms of creatine that do not significantly increase plasma creatine above normal fasting levels (i.e., about 25 µmol/L or 3.75 µg/mL) would have no effect on muscle creatine content because it would not deliver any creatine to tissues for uptake by creatine transporters. Likewise, sources of creatine that increase plasma creatine levels by less than 100 µmol/L (15 µg/mL) would not be considered viable sources of creatine for dietary supplements because they would not deliver enough creatine to target tissue to significantly increase creatine content [12,45]. Viable sources of creatine in dietary supplements should increase plasma creatine levels above 200–500 µmol/L (30–75 µg/mL) within the first hour of oral ingestion and promote a large increase in the AUC of creatine over a 4-to-5 h period [11,12,45]. However, an increase in plasma creatine alone does not provide definitive proof that a purported source of creatine is bioavailable or effective. Differences in plasma creatine after ingestion of a bioavailable source of creatine only suggests that absorption rates may differ. Higher blood creatine could mean that the source is not taken up as quickly into tissue, while lower levels could mean that less appears in the blood or creatine absorption into tissue is faster [48]. Ultimately, the source of creatine must be transported into tissues by creatine-specific transporters and increase tissue levels of creatine in physiologically significant amounts to affect creatine-related metabolism (i.e., 20–40%). Thus, it cannot be assumed that a purported source of creatine will be effectively transported into muscle based on solubility properties in fluid and/or changes in blood concentrations alone because sources of creatine that are not effectively transported into tissue could have higher plasma levels than those that promote a more rapid transport into the tissue. Consequently, it is important to assess the difference between arterial content (amount of creatine delivered to tissue) and venous creatine content or measure the amount of creatine retained in tissues directly to determine the amount of orally ingested creatine taken up by tissue.

#### 3.1.3. Assess Changes in Tissue Creatine Content

Thus, the third step in verifying the bioavailability of a purportedly novel source of creatine is to directly assess the effects of oral ingestion at recommended dosages on tissue creatine content. This is most often done by determining changes in muscle creatine content since 95% of creatine is stored in skeletal muscle. So-called forms of creatine that have been marketed as creatine but do not have any data showing the source of creatine increases muscle creatine content in humans should not be considered a viable source of creatine until such data are available. Purported sources of creatine that increase blood levels of creatine but do not increase tissue levels of creatine in physiologically effective amounts (i.e., 20–40% in muscle and 10–20% in brain) are not bioavailable sources of creatine. Purported sources of creatine that do not deliver similar increases in muscle creatine content than equivalent doses of CrM are less bioavailable sources of creatine than CrM. Moreover, purported derivatives or analogs of creatine that have no measurable effects on plasma creatine levels and do not increase muscle creatine content are not bioavailable sources of creatine, and therefore could have no physiological effects that have been reported in the literature from CrM supplementation. Likewise, if a form of creatine does not significantly increase muscle and/or brain creatine content in humans at the recommended dosages, it should not be considered a viable source of creatine for a dietary supplement. This includes making claims that lower doses of a purportedly more bioavailable source of creatine (e.g., 1–2 g) or “sprinkling” physiologically insignificant amounts of CrM or other purported creatine sources, derivatives, or analogs of creatine in supplements or beverages (e.g., 25–250 mg) promote similar or better benefits as CrM loading (e.g., 4 × 5 g/day for 5–7 days) or long-term supplementation (e.g., 3 g/day).

## 4. Physio-Chemical Properties

**Figure 3:**
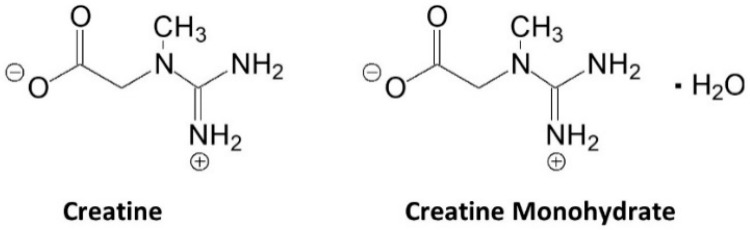
Chemical structure of creatine and creatine monohydrate.

Figure 3 shows the structure of creatine and CrM. Creatine monohydrate was the first source of creatine marketed as a dietary supplement and remains the most common source of creatine found in dietary supplements [25]. Creatine monohydrate is considered the gold standard to compare other purported sources of creatine because of its well-known physiochemical properties, high bioavailability, stability, low cost, and a large number of studies that have demonstrated efficacy and safety [15,25]. CrM has been so extensively studied compared to other purported forms of creatine that when creatine supplementation is discussed in the literature, it is understood the authors are referring to CrM unless otherwise specified [15,17,18,49]. Nevertheless, CrM is formed by crystallization with water forming monoclinic prisms that hold one molecule of water per molecule of creatine [25]. This provides a powder containing 87.9% creatine that readily dissociates into creatine and water upon oral ingestion. The water in CrM can also be removed when exposed to heat at about 100 °C yielding anhydrous creatine that is 100% creatine [25]. However, due to the increased temperature used during the drying, anhydrous creatine contains higher amounts of the impurity creatinine. Creatine appears as internal salt and is considered a fairly weak base (pKb 11.02 at 25 °C) that forms salts with strong acids (i.e., pKa < 3.98) [25]. Creatine can also complex with other compounds via ionic binding (i.e., the attraction of positive cation and negative anion charges).

Pischel and Gastner [50] described the basic process of industrial synthesis of CrM. The process involves adding acetic acid to an aqueous sodium sarconsinate solution and stirring to a pH value of about 10 and a temperature of about 80 °C. An aqueous cyanamide solution is then added to the medium and agitated to facilitate the reaction. After cooling, the crystalline CrM is filtered, separated, and then dried [50]. Creatine monohydrate is manufactured by using water as solvent in Germany has produced 99.9% pure CrM with no contaminants under the brand name Creapure^®^. Other sources of CrM, particularly from China, have been reported to contain contaminates like dicyandiamide, dihydrotriazine, dimethyl sulphate, thiourea, creatinine, and/or higher concentrations of heavy metals like mercury and lead due to the use of different chemical precursors, poorly controlled synthesis processes, using organic solvents, and/or less than adequate filtration methods that increase production of these contaminants [50]. For this reason, creatine monohydrate manufactured by AlzChem in Germany is considered the gold standard of creatine and has been the primary source of creatine used in hundreds of clinical trials conducted on CrM to establish safety and efficacy [15,25].

## 5. Stability

**Figure 4:**
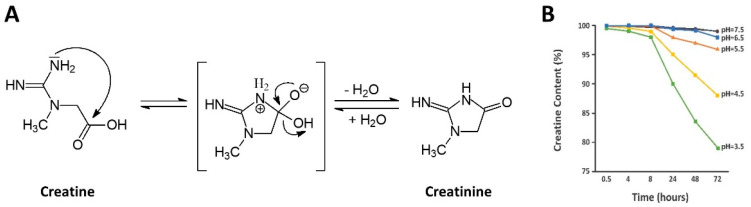
Conversion of creatine to creatinine (**A**) and influence of pH on creatine stability in solution (**B**). Creatine stability figure adapted from Howard and Harris [55].

Creatine monohydrate is very stable in powder form, showing no signs of degradation to creatinine over years, even at elevated temperatures [25]. For example, Jäger [51] reported that CrM powder showed no signs of degrading to creatinine even with temperatures up to 40 °C (104 °F) for more than three years. Additionally, even when CrM was stored at 60 °C (140 °F), creatinine could only be detected in trace amounts after 44 months of storage [51]. However, creatine is not as stable in solution due to intramolecular cyclization that converts creatine to creatinine (see Figure 4A). Generally, creatine degrades to creatinine in solution at a faster rate as pH decreases and temperature increases [25,52,53,54]. For example, as seen in Figure 4B, Harris and coworkers [55] reported that creatine is relatively stable for 3 days in solution at neutral pH (6.5 to 7.5) However, the rate of degradation to creatinine increased when stored at 25 °C when pH decreased (e.g., 4% at 5.5 pH; 12% at 4.5 pH; and 21% at 3.5 pH) [55]. However, as described below, the conversion of creatine to creatinine is halted at pH levels < 2.5. For this reason, it is recommended that CrM should be consumed immediately after it is mixed in an acidic beverage or refrigerated to slow the degradation to creatinine and consumed within a couple of days. However, recent reports presented shelf-life stability data of CrM suspended in a solution of 70% for 13-months at neutral pH and 100% for 12 months at a pH of 2.8 [56,57].

**Figure 5:**
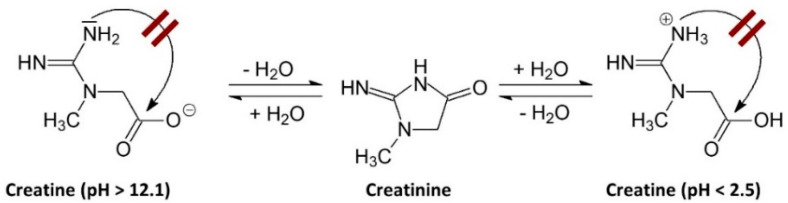
Influence of pH on creatine stability in solution.

As mentioned above, the degradation of creatine can be limited or prevented when creatine is in very low or very high pH environments [25]. In this regard, a pH > 12.1 promotes the deprotonation of the acid group. This makes it more difficult for intramolecular cyclization to form creatinine [25]. On the other hand, when pH is <2.5, the amide functional group on the creatine molecule is protonated and prevents the intramolecular cyclization (see Figure 5) [25]. Since stomach acid is generally less than 2.5, less than 1% of CrM is degraded to creatinine during digestion and 99% of creatine is taken up by tissue or excreted in urine after ingestion [12,25,58,59].

## 6. Solubility

One of the limitations in terms of developing consumer products containing CrM is that CrM powder is not highly soluble. For example, when mixing CrM in solution, some CrM residue remains at the bottom of the glass requiring consumers to add more fluid, swirl, and quickly ingest to ensure they consumed all the creatine. While this has no effect on creatine bioavailability as CrM is nearly 100% bioavailable [12,25,58,59], there has been interest in finding ways to improve the solubility of creatine. The solubility of creatine in water increases linearly with increasing temperature. In this regard, about 6 g of creatine dissolves in one liter of water at 4 °C (39.2 °F) while 14 g/L are dissolved at 20 °C (68 °F), 34 g/L are dissolved at 50 °C (122 °F); and, 45 g/L are dissolved at 60 °C (140 °F) [25]. This is the reason that some researchers initially administered CrM to participants in warm to hot water [12] or hot tea [60]. Creatine solubility can also be improved by administering CrM in lower pH solutions like juices and sport drinks that generally have pH levels ranging from 2.5–3.5 [61] and/or blending CrM with carbohydrate and/or protein powders or in juice which helps suspend CrM in solution, reduce sedimentation, and enhance creatine uptake into muscle [20,21,22,23,62].

Dissolving creatine in more acidic environment is also the rationale in providing creatine in the form of easily disassociated creatine salts. Adding an acidic moiety to a creatine molecule lowers the pH of the water. For example, adding tri-creatine citrate to water yields a pH of 3.2 and increases solubility to 29 g/L whereas adding creatine pyruvate to water yields a pH of 2.6 and increases solubility to 54 g/L [63]. Creatine hydrochloride (HCl) has also been marketed as a pH lowering source of creatine with greater solubility than CrM [64,65]. While lowering pH and/or mixing creatine salts into solution enhances solubility, the amount of creatine contained in these forms of creatine salt must be equalized to CrM to deliver the same amount of creatine to the blood and tissues. In this regard, CrM contains 87.9% creatine whereas creatine citrate (40.6%), di-creatine citrate (57.7%), creatine pyruvate (59.8%), and creatine HCl (78.2%) contain less creatine by weight compared to CrM. Therefore, one would need to mix 1.54, 1.34, 1.32, and 1.11 times more of these forms of creatine in solution to deliver the same amount of creatine than CrM. Additionally, while mixing CrM in common juices and beverages with pHs ranging from 2.5–3.5 [61] would enhance solubility, it would also promote the conversion of creatine to creatinine over time [66]. Therefore, it is best to consume creatine salts or CrM with acid beverages soon after it is mixed so that the conversion of creatine to creatinine would be halted upon entering a more acidic environment in the stomach.

With that said, the only real advantage of mixing CrM in an acidic beverage is that it would leave less CrM in crystalized form at the bottom of a cup to swirl and consume during the last drink. If an individual consumes all the CrM (soluble or not), it will be bioavailability in terms of intestinal absorption, transport of creatine in the blood, transport of creatine through tissue-specific creatine transporters, and uptake of creatine into tissues. Similarly, if a bioavailable source of creatine is consumed at physiologically effective doses, it is not degraded during digestion, and it increases blood and tissue creatine content by physiologically meaningful amounts (i.e., 20–40%), it does not matter whether a form of creatine has better mixing characteristics and/or is more soluble. Research has clearly shown that CrM is not degraded into creatinine during normal digestion, it is nearly 100% bioavailable [12,25,58,59], and markedly increases blood and tissue creatine content. There are no data showing that any other purported form of creatine is more effective in increasing tissue creatine content than CrM [62]. Therefore, claims that a given form of creatine is more effectively absorbed than CrM because it is more soluble in water is unsupported marketing hyperbole.

## 7. Purported Creatine Related Compounds

Table 1 provides a list of creatine-related compounds that are listed in PubChem when doing a search on the word creatine. As of this writing, 88 creatine-related compounds are listed in the PubChem database. A few others that have been mentioned in patents, published literature, or on international regulatory authorities’ lists are also mentioned at the bottom of Table 1. While not all these compounds have been used in dietary supplements or assessed for bioavailability, it provides an overview of the types of creatine-related compounds that have been developed. Interestingly, some creatine derivatives and analogs that have been marketed as bioavailable sources of creatine in dietary supplements are not listed in this database because the molecular structures have been altered such that the compound does not contain the creatine molecule. Consequently, they should not be considered a bioavailable source of creatine unless studies show that it increases creatine levels in the blood and target tissue (i.e., muscle and brain). This view is consistent with the United States FDA definition that a dietary supplement is considered a new dietary ingredient (NDI) if the ingredient has been chemically altered from its natural form [67].

It should be noted that different creatine-related compounds shown in Table 1 contain less creatine by molecular weight than CrM, assuming that a full creatine molecule is contained in the compound and would be liberated as creatine in circulation. Consequently, it would take a greater amount of most of these sources of creatine in a dietary supplement to provide equivalent amounts of creatine delivered from CrM if the other sources were indeed bioavailable. For example, creatine citrate (CC) contains 53.9% less creatine by molecular weight than CrM. Therefore, a supplement would need to provide 9.3 g of CC to equal a typical supplementation dose of 5 g of CrM if it had similar bioavailability. Creatine ethyl ester (CEE) has only 6.3% less creatine by molecular weight than CrM. However, as will be discussed below, some of the creatine in CEE converts to creatinine during digestion and therefore it is less bioavailable than CrM. Therefore, more CEE would have to be provided in a supplement to provide an equivalent amount of creatine to tissue and it may increase serum creatinine levels to a greater degree. Additionally, if a purported source or derivative of creatine does not break down and increase blood creatine levels and creatine content in muscle, it would not be a bioavailable source of creatine no matter how much was in the supplement. These are important points to consider when developing nutritional formulations or conducting research with these other sources of creatine.

## 8. Strong Evidence to Support Bioavailability, Efficacy, and Safety

### Creatine Monohydrate

As noted above, CrM is the gold standard to compare other purported forms of creatine due to its known bioavailability, pharmacokinetics, efficacy, and safety [5,14,15,25,62]. Prior studies indicate that CrM loading (i.e., 4 × 5 g/day for 5–7 days) or low-dose long-term intake (e.g., 3–6 g/day for 4–12 weeks) increases muscle creatine retention typically by 20–40% depending on initial creatine content in the muscle [12,22,68,69,70,71] and brain creatine content by 5–15% [72,73,74,75,76,77]. CrM supplementation has been reported to improve acute exercise performance particularly in intermittent high-intensity exercise bouts as well as enhance training adaptations in adolescents [78,79,80,81,82], young adults [29,55,83,84,85,86,87,88,89,90,91,92], and older individuals [8,77,93,94,95,96,97,98,99,100,101]. High-intensity exercise performance is generally increased by 10–20% with greater improvements seen in individuals starting the supplementation protocol with lower muscle creatine and PCr content [102]. Improvements in performance have been reported in individuals participating in weight training [55,89,95,103,104,105,106,107,108,109,110,111,112,113], running [114,115,116,117,118], soccer [87,119,120], swimming [79,80,121,122,123,124], volleyball [125], softball [126], ice hockey [127], golf [128], among others [24]. Men and women have been reported to benefit from CrM supplementation in populations ranging from children to elderly populations [47,80,119,126,129,130,131,132,133].

Uptake of creatine into muscle with CrM supplementation has been reported to be enhanced when CrM is consumed with carbohydrate [20,21,22] and carbohydrate and protein [23]. Co-ingesting CrM with nutrients that improve insulin sensitivity like D-pinitol [134], Russian Tarragon [135], and Fenugreek extract [136] have been reported to enhance creatine retention with limited to no additive effects on exercise performance or training adaptations [89,136,137]. There is also no evidence that supplementation of micronized versions of CrM (i.e., CrM with smaller mesh size particles) are more bioavailable than CrM with normal mesh sized particles [138,139,140] or consuming CrM in effervescent fluid promotes greater creatine retention or performance benefits [22,141]. This makes sense in that while small differences in creatine retention may enhance the rate of saturating creatine stores (e.g., in 4 versus 5 days) or ensure more consistent response to CrM supplementation (e.g., consuming creatine with carbohydrate and/or carbohydrate and protein), once creatine stores are fully saturated, there would be no ergogenic or training advantage. No side effects have been reported with CrM supplementation other than a desired weight gain [15]. Additionally, there is no convincing evidence that CrM causes common anecdotal myths like bloating, gastrointestinal distress, disproportionate increase in water retention, increased stress on the kidneys, increased susceptibility to injury, etc. [62,142,143,144]. Many of these claims have been described in marketing materials by companies attempting to gain market share for their purported creatine-containing products [25,62].

Based on a large body of evidence, the ISSN concluded that CrM is the most effective ergogenic nutrient currently available to athletes in terms of increasing high-intensity exercise capacity and lean body mass during training [8,15]. Position stands by the American Academy of Nutrition, Dietitians of Canada, and the American College of Sports Medicine on nutrition and athletic performance [17,49]; the International Olympic Committee consensus statement on dietary supplements and the high-performance athlete [18]; and, the Office of Dietary Supplements, Dietary Supplement Fact Sheets on Dietary Supplements for Exercise and Athletic Performance [145] provided similar conclusions. Thus, there is consensus among professional organizations that CrM is an effective nutritional ergogenic aid that may benefit athletes involved in numerous sports and individuals initiating exercise training to promote health and fitness.

Given the metabolic and ergogenic properties of CrM [7,14,15,24], there has also been interest in assessing the effects of CrM supplementation in various clinical populations that may benefit from increasing high-energy phosphate availability and/or increasing strength and muscle mass [5]. A recent Special Issue on creatine supplementation for health and clinical diseases overviewed the metabolic basis of creatine in health and disease [5] and potential health and/or therapeutic benefits of CrM supplementation for pregnancy and newborn health [146], children and adolescents [147,148,149,150,151], physically active young adults [24], rehabilitation [152], women’s health [133], older adults [44], brain health and cognitive function [74], glucose management and diabetes mellitus [153], immunity [154], T cell antitumor immunity and cancer therapy [155], heart health [156], vascular health [157], inflammatory bowel disease [158], chronic renal disease management [159], and post-viral fatigue [160]. From what we can see, all studies in these populations used CrM as the source of creatine. Thus, there is substantial evidence to support the safety and efficacy of CrM supplementation (see Table 2). Additionally, this body of evidence provides the basis to compare the efficacy and safety of other purported sources of creatine. For this reason, CrM is classified as having strong evidence from pharmacokinetic and tissue bioavailability studies, numerous randomized and controlled clinical trials, and a long-history of safety assessed in clinical trials and historical widespread use supporting bioavailability, efficacy, and safety.

## 9. Some Evidence to Support Bioavailability, Efficacy, and Safety

### 9.1. Creatine Salts

Creatine salts were introduced into the marketplace in the early 1990s and are formed by adding an acid moiety to creatine, complexing an acid to the creatine molecule, or adding an acid to a complexation product [25]. The rationale was to combine creatine with acids that could easily dissociate (e.g., ionic bond) upon ingestion, thereby not only serving as a viable way to deliver creatine to tissue, but also deliver other nutrients that may have ergogenic properties and/or promote a synergistic metabolic effect with creatine. Additionally, to find ways to improve physical characteristics like solubility of creatine. To do so, the creatine salt must deliver physiologically effective doses of creatine (i.e., 3–5 g per serving) to the blood and tissue in an equivalent manner as CrM to be comparatively effective. Additionally, the acid added to creatine would have to provide a more synergistic effect than simply co-ingesting CrM with the acid independently in a nutritional formulation. Finally, the theoretically added benefit must justify the additional expense in producing the creatine salt and including it in a nutritional formulation.

A number of creatine salts have been marketed as sources of creatine for dietary supplements including creatine citrate (including di- and tri- forms) [172,173,174]; creatine maleate, creatine fumarate, creatine tartrate [31]; creatine pyruvate [32,174,175,176]; creatine ascorbate [33]; and creatine orotate [30,176,177], among others. Some creatine salts are less stable when compared to CrM. For example, storage of tri-creatine citrate at 40 °C (104 °F) for 28 days results in formation of 770 ppm of creatinine compared to no measurable amount with CrM powder [63]. Adding carbohydrate to the formulation has been reported to improve the stability of some creatine salts [178]. However, creatine salts would also have less stability than CrM in solution since adding the acid to creatine decreases pH to ranges that would promote greater formation of creatine to creatinine in solution over time. The following summarizes results of studies that provide some evidence of bioavailability, efficacy, and safety of creatine salts.

#### 9.1.1. Creatine Citrate

**Figure 6:**
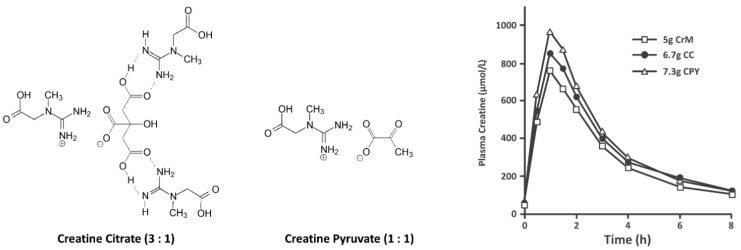
Chemical structure of tri-creatine citrate and creatine pyruvate with plasma creatine changes after oral administration of equal molar doses of creatine monohydrate (CrM), creatine citrate (CC) and creatine pyruvate (CPY). Adapted from Jäger et al. [63].

Figure 6 shows the chemical structure of two common creatine salts. Jäger et al. [63] compared the effects of oral ingestion of 5 g of CrM to 6.7 g of tri-creatine citrate (CC) and 7.7 g of creatine pyruvate (CPY) that provided equimolar amounts of creatine. Tri-creatine citrate is a 1:1 salt of creatine and citric acid, with two additional creatines forming a complex with the 1:1 salt. The second and third acid moiety of citric acid are not strong enough acids to form salts with creatine. Peak concentrations of creatine were significantly higher with CPY (CrM 761.9 ± 107.7, CC 855.3 ± 165.1, CPY 972.2 ± 184.1 µmol/L) while AUC values did not significantly differ among treatments (2384 ± 376.5, 2627 ± 506.8, 2985 ± 540.6 mM/h, respectively). Results support contentions that provision of equimolar amounts of CC and CPY can serve as a bioavailable source of creatine. Conversely, Gufford et al. [179] reported that CC and CPY had less permeability than CrM across caco-2 monolayers cells, which is used as a model to assess intestinal absorption. However, it is unlikely that the small differences in blood creatine levels observed would promote greater creatine retention and/or a bioenergetic advantage. To date, we are unaware of any study that has evaluated the effects of CC or CPY on muscle or brain creatine content.

In terms of efficacy, several studies have evaluated whether creatine citrate (CC) supplementation can affect exercise capacity. For example, Smith and colleagues [180] found ingesting 20 g/day of di-creatine citrate for 5 days delayed neuromuscular fatigue in women. Jäger et al. [174] reported that ingestion of 5 g/day of CC (providing 3.25 g/day of creatine) for 28 days significantly increased intermittent maximal effort handgrip force compared to placebo. Graef and coworkers [181] reported that 10 g/day of CC supplementation for 30 days during high-intensity interval training significantly increased the ventilatory threshold, but did not enhance maximal aerobic capacity. Smith and colleagues [172] reported that 20 g/day of creatine di-citrate supplementation for 5 days had no detrimental or ergogenic effects on running critical velocity, aerobic capacity, or time to exhaustion. Finally, Fukuda and assistants [182] reported that CC supplementation (4 × 5 g with 18 g dextrose for 5 days) improved anaerobic run capacity in men, but not women. While these studies provide evidence that CC can serve as a viable source of creatine by increasing blood creatine level in a similar manner as CrM and there is some data supporting the ergogenic benefit compared to placebo, the impact of CC supplementation has not been assessed on muscle or brain creatine content. Therefore, there are no studies indicating that CC is more bioavailable, more effective, or a safer source of creatine than CrM. Given this, CC is categorized as having limited evidence to support bioavailability, efficacy, and safety.

#### 9.1.2. Creatine Pyruvate

**Figure 7:**
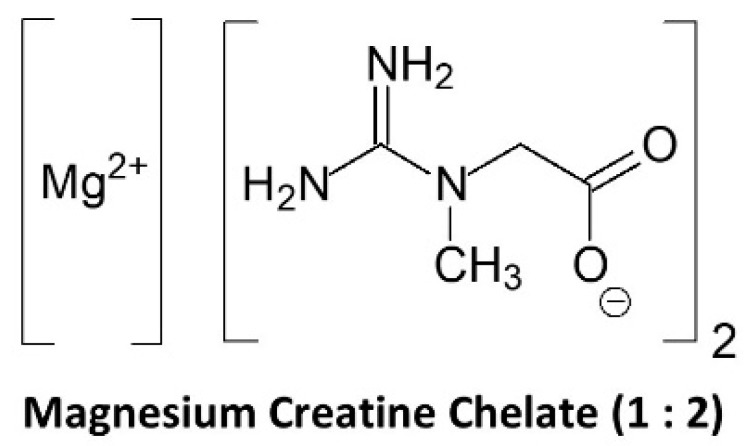
Chemical structure of magnesium creatine.

Calcium pyruvate supplementation (e.g., 6–25 g/day) has been reported to affect exercise performance and promote fat loss [183,184,185,186]. Stone et al. [83] also reported that CrM and the combination of CrM and calcium pyruvate supplementation during 5 weeks of off-season training improved training and body composition adaptations. In contrast, calcium pyruvate supplementation alone had no effects. Therefore, there was some rationale in developing a creatine salt with pyruvate [32]. As seen in Figure 7, ingestion of 7.3 g of CPY promoted significant increases in plasma creatine levels in a similar manner as ingesting 5 g of CrM [63]. This group also reported that ingestion of 5 g/day of CPY (providing 3 g/day of creatine) for 28 days significantly increased intermittent maximal effort handgrip force [174]. Another study found that ingesting 7 g/day of CPY for 7 days had no effects on endurance capacity or repeated sprint performance in cyclists [175]. However, ingesting 7.5 g/day of CPY improved paddle rate and lowered blood lactate in Olympic canoeists suggesting an improvement in aerobic exercise efficiency [187]. These studies indicate that CPY can serve as effective source of creatine to increase blood creatine content and a few short-term studies suggest there may be some ergogenic values. However, since there are no data assessing the effect of CPY supplementation on muscle or brain creatine content and only a limited number of studies have evaluated efficacy and safety, CPY is classified in the limited bioavailability, efficacy, and safety category. With that said, there is no evidence that CPY is more effective than CrM in increasing muscle creatine content and/or performance.

### 9.2. Magnesium Creatine Chelate

Magnesium creatine chelate (MgCr-C) has been marketed as a more bioavailable source of creatine (see Figure 7). The rationale is that since magnesium is a cofactor in ATP reactions and the only mineral that decreases during exercise, there may be additive benefit in combining creatine with magnesium. There are also claims that MgCr-C supplementation can improve muscle protein synthesis. A patent described bioavailable chelates of creatine and essential metals [188]. Since MgCr-C contains about 84.4% of creatine by molecular weight (see Table 1), it could theoretically serve as a good source of creatine if creatine easily dissociates from MgCr-C and equimolar amounts of creatine were consumed compared to CrM. However, it is marketed as a much more bioavailable source of creatine than CrM with recommended doses of only 1 g per 18.2 kg (40 lbs.) of body weight per day (about 3.8 g/day for a 70 kg individual). We are aware of no data showing that MgCr-C increases blood creatine levels or promotes greater creatine retention in skeletal muscle. Thus, there are no data supporting that MgCr-C is more bioavailable to tissue than CrM.

Several studies have evaluated the effects of MgCr-C on performance related variables. For example, Brilla and coworkers [189] evaluated the effects of ingesting 5 g of CrM with 800 mg of magnesium oxide or magnesium plus 5 g of MgCr-C for 2 weeks compared to placebo on body water and isokinetic strength performance in recreationally active participants. Results revealed that body water increased with MgCr-C, but not CrM while torque and power increased similarly with CrM and MgCr-C. Selsby and colleagues [190] evaluated the effects of supplementing 2.5 g/day of CrM, MgCr-C, or a placebo for 10 days on strength and muscle endurance. Results revealed that CrM and MgCr-C were both effective in increasing performance with no differences observed between types of creatine ingested. Finally, Zajac et al. [191] reported that 5.5 g/day of MgCr-C supplementation during 16 weeks of soccer training improved repeated sprint ability performance compared to placebo. Interestingly, creatinine levels were also significantly increased throughout training with MgCr-C (0.83 to 1.87 mmol/L) compared to placebo (0.92 to 0.82 mmol/L), which is higher than reported in other long-term CrM studies during training in hot and humid environments that administered 5–10 g/day of CrM for 21 months in well-trained athletes [144]. However, this study did not compare the effects of consuming MgCr-C to CrM, and performance changes were consistent with other studies conducted on CrM supplementation. Consequently, there is no data showing that MgCr-C increases blood or muscle creatine and there is only limited data showing potential ergogenic effect. There is also no evidence that MgCr-C is more bioavailable, efficacious, and/or a safer source of creatine than CrM. For this reason, MgCr-C is listed in some evidence to support bioavailability, efficacy, and safety category.

### 9.3. Creatine Ethyl Ester

**Figure 8:**
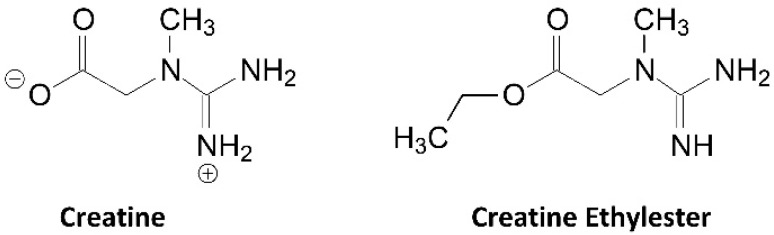
Chemical structure of creatine and creatine ethyl ester.

Another source of creatine that claims to have better solubility, bioavailability, and efficacy than CrM is creatine ethyl ester (CEE). CEE is basically a creatine molecule with a H+ removed from the second N position (i.e., NH versus NH_2_) and a methyl group (CH_2_-CH_3_) added to the terminal O position through an esterification reaction (see Figure 8). Thus, CEE is a chemical alteration of creatine and is not actually creatine. For CEE to act like creatine it would have to be de-ethylated and an H+ added back to the NH of the molecule at nearly 100% efficiency to deliver 94% of an equivalent CrM dose. Marketing claims suggest that CEE is absorbed faster and more efficiently than CrM, so no loading dose is needed. Additionally, CEE is claimed to have less anecdotal side effects than CrM like bloating and dehydration. For this reason, the recommended dosages of CEE are typically 2–6 g/day.

**Figure 9:**
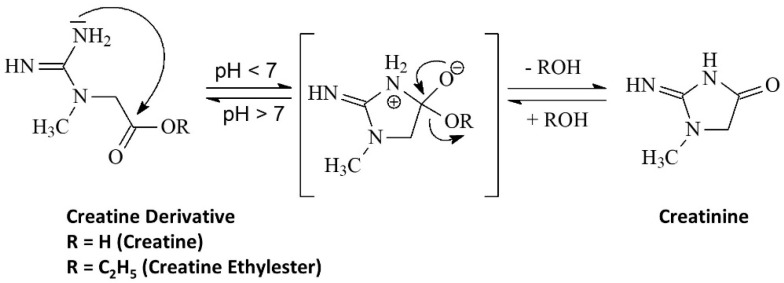
Degradation of creatine ethyl ester to creatinine. Adapted from Jäger et al. [25].

While proponents of CEE have assumed that all orally ingested CEE is converted to creatine in vivo, available studies suggest it is less efficient. For example, Childs and Tallon reported that CEE rapidly degrades to creatinine when exposed to stomach acid [34]. Giese et al. [35,192] reported that under physiological conditions, CEE non-enzymatically converted to creatinine with no measurable conversion to creatine (see Figure 9). Likewise, Katseres and colleagues [193] reported that the half-life of CEE was in the order of one minute suggesting CEE may hydrolyze too quickly to reach muscle cells in its ester form. On the other hand, Gufford et al. [194] reported that CEE converted to creatinine in a linear manner as pH levels dropped below 8.0 and that CEE was mostly stable at a pH of 1.0. Since acidity in the stomach generally ranges from 1.5 to 3.5, it is likely that some CEE is degraded into creatinine during normal digestion while delivering some level of creatine to blood.

**Figure 10:**
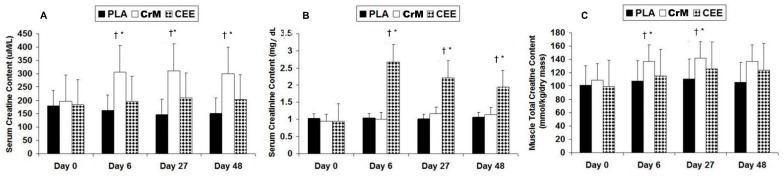
Serum creatine (**A**), serum creatinine (**B**), and muscle creatine content (**C**). * Represents significant change from baseline. † in panel A indicates significantly higher serum creatine concentrations in CrM when compared to PLA (*p* = 0.007) and CEE (*p* = 0.005). † in panel B indicates serum creatinine in the CEE group was greater than PLA (*p* = 0.001) and CrM (*p* = 0.001). † in panel C shows the PLA group was significantly less than the CrM (*p* = 0.026) and CEE (*p* = 0.041) groups. Adapted from Spillane et al. [27].

Spillane and colleagues [27] compared the effects of supplementing the diet with a 0.30 g/kg of fat-free body mass (approximately 20 g/day) for 5 days followed by ingestion of 0.075 g/kg of fat free mass (approximately 5 g/day) for 42 days of a placebo, CrM, or CEE on muscle creatine content and performance adaptations. If CEE is bioavailable, those taking CEE should have increased muscle creatine content better than those taking a placebo. Likewise, if CEE was more bioavailable than CrM, greater changes would be seen in the CEE group compared to those in the CrM group. As seen in Figure 10A, fasting serum creatine levels significantly increased only in the CrM group. While this was not a pharmacokinetic oral dose study, it is interesting that CEE supplementation had no effect on fasting serum creatine levels compared to a placebo. Conversely, CEE significantly increased serum creatinine levels by more than two-fold after 6, 27, and 48 days of supplementation in comparison to the placebo and CrM groups (Figure 10B). The values observed exceeded normal creatine values even for highly trained athletes training in hot and humid environments [13]. In addition, while CEE supplementation promoted a significant increase in muscle total creatine content after 27 days of supplementation compared to those ingesting a placebo, those taking CrM observed significantly greater increases compared to the placebo and CEE groups (Figure 10C). These findings suggest a large amount of CEE is converted to creatinine and CEE is less effective in increasing muscle creatine content than CrM. This was despite including a 20 g/day loading dose of CEE that manufacturers claimed is unnecessary due to greater bioavailability. Moreover, CEE supplementation did not promote greater changes in body composition, strength, or anaerobic power during training compared to CrM supplementation. These findings directly refute claims that CEE is more bioavailable source of creatine than CrM and that CEE promotes greater training adaptations than CrM. Further, the clinically significant increase in creatinine levels observed should raise some concerns about potential safety of CEE as has reported two case studies [195,196].

With that said, some have pointed to the results of a recent study conducted by Arazi and associates [197] to support claims about the efficacy of CEE. The researchers evaluated the effects of 6 weeks of CEE supplementation (20 g/day for 5 days and 5 g/day for 37 days) compared to consuming a placebo during resistance training (3 sets of 8–10 repetitions at 60–80% or one repetition maximum, 3 times per week) in untrained, younger, and underweight men. The investigators reported that CEE supplementation during resistance-training promoted significant increases in body weight and leg press strength, while percent body fat decreased to a greater degree with placebo ingestion. In addition, some differences were reported in anabolic and catabolic hormones that prior have not been previously studies conducted on creatine had not reported [62]. While this study indicates that higher than recommended doses of CEE can positively affect training adaptations, this study did not assess the effects of CEE supplementation on blood or muscle creatine content or compare the efficacy of CEE supplementation to CrM. The results observed are consistent with those found in the Spillane et al. study [27] in that CEE had some benefit over placebo ingestion, but results were not better than CrM. Given that CEE promoted a modest but less effective increase in muscle creatine content in that study, one would expect some benefit of CEE supplementation during training if higher than recommended doses are ingested. However, there is no evidence that ingesting recommended doses of CEE is effective or that ingesting typical CrM loading, and maintenance doses of CEE is more effective than CrM. We are also not aware of any studies that have evaluated other marketed forms of CEE (i.e., creatine methyl ester hydrochloride, di-acetyl creatine ethyl ester, creatine ethyl ester pyruvate, creatine ethyl ester malate, or creatylglycine ethyl ester fumarate). Nevertheless, since there is some evidence that ingesting high doses of CEE can increase muscle creatine content and performance compared to placebo, we have categorized CEE in some evidence category. However, we recommend that additional research evaluate safety given the increased creatinine levels observed.

### 9.4. Creatine HCl

**Figure 11:**
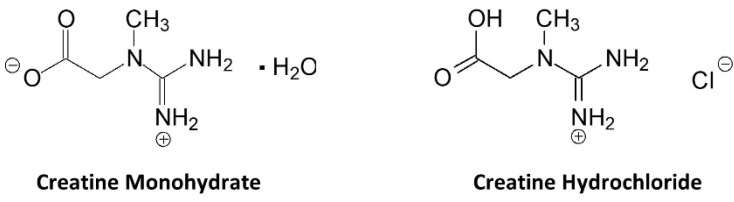
Comparison of creatine monohydrate and creatine HCl structures.

Creatine hydrochloride (Cr-HCl) has been marketed as a more bioavailable source of creatine than CrM. As shown in Figure 11, Cr-HCl is a salt of HCL and creatine molecule. Like other creatine salts, adding hydrochloric acid to creatine would be expected to decrease pH and improve solubility. Marketing claims indicate that Cr-HCl has a 38 times greater bioavailability than CrM [198]. The basis for this claim appears to come from a report from Gufford and colleagues [179] who conducted physiochemical characterization studies on several N-methylguanidinium salts, including creatine Cr-HCl. They reported that Cr-HCl contains about 78% creatine by molecular weight and that Cr-HCl was 37.9 times more soluble in water than CrM at 25 °C. However, CrM was assessed at a saturation pH of 8.6 while Cr-HCL was measured at a saturation pH of 0.3. While mixing creatine in acidic solutions may improve solubility when mixed in water, as noted above, it would have no effect on bioavailability. These claims are also apparently based on a report from Alraddadi et al. [199] who conducted a bioavailability study in rats with labeled creatine (creatine-^13^C) at a low (10 mg/kg) and high (70 mg/kg) oral doses. They then assessed the amount of creatine-^13^C incorporated into plasma, muscle and brain tissue and used a simulated prediction model to estimate how Cr-HCl would theoretically affect tissue creatine retention based on differences in solubility. While this is an interesting approach, there are several problems with using these findings to make claims about Cr-HCl. First, this is only a theoretical modeling study. The researchers did not directly compare Cr-HCl to CrM intake on plasma or tissue creatine content. Second, it is well-known that there are species specific differences in creatine metabolism and storage [200]. Therefore, you cannot directly extrapolate results from mice or rat data to human creatine oral dosing studies. Pharmacokinetic and creatine retention studies need to be conducted in humans to assess whether Cr-HCl promotes greater creatine retention in tissue to assess the validity of this claim.

As of this writing, no PubMed indexed articles have been published on Cr-HCl and muscle creatine retention or performance. However, several articles have been published in non-indexed journals from Brazil that have been cited in marketing materials. In the first study, de França and colleagues [65] evaluated the effects of supplementing the diet with 1.5 g/day of Cr-HCl, 5 g/day of Cr-HCl, and 5 g/day of CrM compared to controls during 4 weeks of resistance-training in 40 young recreational weightlifters on strength gains and skinfold caliper determined body composition. The researchers reported some benefits of Cr-HCl and CrM supplementation on leg press and skinfold determine body composition. However, the use of skinfold calipers to estimate body composition and statistical analysis methods employed make it difficult to draw any conclusions. In fact, gains in fat-free mass were greatest in the CrM group (+1.7 kg), but supposedly not significantly different than observed with 5 g/day of Cr-HCL (+1.6 kg) that were reported to be significantly different than controls (+1.1 kg) and those ingesting 1.5 g/day of Cr-HCl (+1.1 kg). In a follow-up study [201], this research group administered 5 g/day of CrM or 1.5 g/day of Cr-HCL with 3.5 g/day of resistant starch for 30-days to Brazilian Olympic level athletes. Results revealed both groups increased skinfold caliper determined fat-free mass and strength, although bioelectric impedance determined total body water was increased to a greater degree in the CrM group (CrM + 1.81 L vs. Cr-HCL + 0.24 L). This would be expected, given the creatine content based on molecular weight in these dosages was 35.1 g in the Cr-HCl group compared to 131.9 g in the CrM group over the 30-day period. Finally, a study conducted by Tayebi and Arazi [202] evaluated the effects of ingesting 3 g/day of Cr-HCL, 3 g/day of CrM, and 20 g/day of CrM, or a placebo for 7 days on anaerobic power and hormone levels. Results revealed that ingestion of 3 g/day of Cr-HCl did not promote greater gains in performance or hormonal responses than 3 or 20 g/day of CrM as claimed. The authors concluded that Cr-HCl does not appear to be a more effective source of creatine than CrM. Thus, while Cr-HCl is a simple salt that should readily disassociate into creatine and HCL, there is no evidence that Cr-HCl is absorbed more effectively than CrM in humans; Cr-HCl promotes greater muscle creatine retention than CrM at equivalent doses; or, that lower doses of Cr-HCl are as effective as standard supplementation protocols with CrM. Given this analysis, Cr-HCl is classified in some evidence category to support efficacy compared to placebo. However, claims that Cr-HCl is more bioavailable, effective, and/or a safer source of creatine than CrM are not supported.

### 9.5. Creatine Nitrate

**Figure 12:**
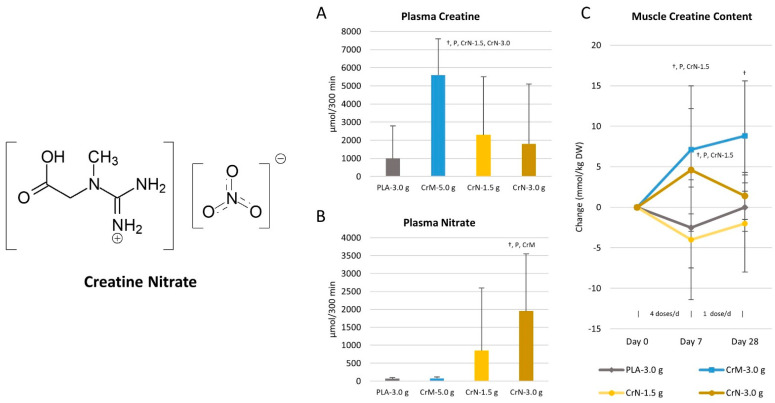
Chemical structure of creatine nitrate. (**A**,**B**) show 5 h area under the curve data after plasma creatine and plasma nitrate, respectively. (**C**) shows mean changes in muscle creatine content with 95% confidence intervals after 7 days of loading 4 doses/day and 21 days of ingesting 1 those/day. ^†^ Represents significant change from baseline, PLA and P represent placebo, CrM represents creatine monohydrate, and CrN represents creatine nitrate.

A number of studies have indicated that dietary nitrates, typically ingested in the form of beet root juice or nitrate powder, can improve endurance [203] and explosive exercise capacity [204]. Recommended dosages generally range between 300–600 mg ingested 1–2 h prior to exercise [204,205,206]. Since nitrate can ionically bond to creatine and form a salt (see Figure 12), creatine nitrate (CrN) has been developed and marketed as a bioavailable source of creatine for dietary supplements [207]. By molecular weight, CrN contains 67.5% creatine. Therefore, ingesting 1 g of CrN would theoretically provide 0.675 g of creatine and 0.325 g of nitrate. Marketing claims suggest greater bioavailability and therefore recommended doses are typically 1–2 g of CrN per/day [208]. In terms of bioavailability, there is limited data available. However, Galvan and colleagues [29] conducted a pharmacokinetic study evaluating the effects of acute oral ingestion of a placebo, 1.5 g of CrN (CrN-Low), 3 g of CrN (CrN-High), and 5 g CrM on blood creatine and nitrate levels. Results revealed that the plasma creatine AUC over a 5 h period for CrM (5634.4  ±  1949.8 μmol/L) was significantly greater than the placebo (1012.4  ±  1882.2 μmol/L), CrN-Low (2342.0  ±  3133.3 μmol/L, *p*  =  0.004), and CrN-High (1761.7  ±  3408.8 μmol/L, *p*  =  0.007) treatments with no differences seen between the CrN dosages. Conversely, the nitrate AUCs in the CrN groups were significantly greater than the placebo and CrM treatments in a dose related manner. These investigators also evaluated the effects of ingesting four doses a day of either a placebo (5 g dextrose), CrM (3 g CRM with 2 g dextrose, CrN-Low (1.5 g CrN, 3.5 g dextrose), and CrN-High (3 g CrN, 2 g dextrose) for 7 days followed by ingesting one dose per day for 21 days as a maintenance dose. Muscle biopsies were obtained at 0, 7, and 28 days to assess muscle creatine content. Results revealed that 7 days of creatine loading (12 g/day of CrM and CrN) significantly increased muscle creatine content in the CrM (7.1 mmol/kg DW) and CrN-High (4.6 mmol/kg DW). However, no difference was seen compared to placebo was observed when ingesting 4 × 1.5 g/day of CrN for 7 days or after 28 days of taking 1.5 or 3 g/day of CrN. These findings suggest that CrN can be a bioavailable source of creatine proportional to the amount of creatine delivered during the loading phase (i.e., 54.6 g for CrN-High versus 73.8 g for CrM), but not more bioavailable than CrM when equivalent doses are ingested. On the other hand, Ostojic et al. [209] conducted a study evaluating the effects of CrM and CrN supplementation on MRS determined skeletal muscle creatine content and markers of health. In a randomized and crossover manner with a 7 day washout period, participants ingested a placebo, 3 g/day of CrN, 3 g/day of CrM, or 3 g/day of CrN + 3 g/day of CrM for 5 days. This theoretically provided a total of 0, 9.75, 13.2, and 22.9 g of creatine during the 5-day period. The researchers found that peak serum creatine increased to a greater degree with CrN + CrM supplementation (CrM 118.6 ± 12.9, CrN 163.8 ± 12.9; CrN + CrM 183.7 ± 15.5 µmol/L) while muscle creatine increased to a greater degree with CrN ingestion (CrM 2.1%, CrN 8.0%, CrN + CrM 9.6%). However, a limitation to this study is that only a 7-day washout period was observed between treatments. It is well known that it takes about 4 weeks for muscle creatine to return to normal after creatine supplementation [47]. Thus, it is possible that the testing order may have confounded results. Nevertheless, results are conflicting on whether short- or long-term CrN supplementation (3 g/day) significantly increase muscle creatine levels.

In terms of performance, Gavan and colleagues found some ergogenic benefit of CrN supplementation on muscular endurance, but they were unrelated to changes in muscle creatine content, suggesting the ergogenic benefit was primarily due to providing ergogenic levels of nitrate and not creatine at the dosages studied. Dalton and colleagues [36] reported that ingesting 3 and 6 g/day of CrN for 5 days significantly improved some measures of strength and muscle endurance compared to placebo. However, it is unclear whether these changes were primarily due to creatine and/or nitrate. Some improvement in exercise performance was also reported with acute [210] and 8-weeks [211] supplementation of a pre-workout supplement containing 2 g/day of CrN. However, since the supplement contained caffeine and other ergogenic nutrients, the benefits cannot be attributed to CrN. Further, it remains to be determined whether CrN supplementation has any additional benefit than simply co-ingesting CrM another source of nitrate (e.g., beet powder).

In terms of safety, since nitrates may lower blood pressure, there has been some concern that CrN may promote hypotension, particularly around intense exercise and/or if individuals take higher than recommended doses. Several studies have assessed safety of acute and chronic CrN supplementation. Dalton et al. [36] reported that ingestion of up to 6 g of CrN for 6 days does not negatively affect resting hemodynamics, response to a postural challenge, the ability to perform high-intensity exercise, or clinical chemistry profiles. Joy and colleagues [212] reported that 28 days of CrN supplementation (1 and 2 g/day) during training had no adverse effects on clinical blood chemistries compared to a non-supplemented group. Galvan and coworkers [29] also found no adverse effects after 28 days of supplementation (3 g/day). Finally, Jung and associates reported no adverse effects of participants consuming a pre-workout supplement containing 2 g/day of CrN for 8 weeks. Thus, CrN appears to be safe when taken in these amounts and timeframes. However, CrN has only been approved as a dietary supplement by the U.S. FDA at levels of 750 mg per day, which is below any meaningful level expected to increase muscular creatine levels and performance. Based on this analysis, there is some evidence showing CrN may serve as a bioavailable and effective source of creatine. However, most studies have used higher than recommended doses, and those studies show that CrN is not more effective than CrM supplementation.

### 9.6. Buffered Creatine Monohydrate

In the early 2010s, a “buffered” or “pH-correct” form of CrM was heavily marketed as a more bioavailable source of creatine than CrM [213]. According to the patent [214], CrM was better stabilized by adding an alkaline powder (e.g., soda ash, magnesium glycerol phosphate, bicarbonate) to CrM (or other purported forms of creatine) in order to increase pH between 7–14. Consequently, they developed CrM that was “synthesized to a pH of 12” (CrM-Alk) and claimed that due to greater stability in preventing the conversion of CrM to creatinine, CrM-Alk was up to 10 times more bioavailable than CrM [213]. Therefore, 1.5 g of CrM-Alk was purported to be equivalent to ingesting 10–15 g of CrM. Additionally, the company theorized that since less CrM-Alk was needed to be ingested, there would be fewer side effects than CrM [213]. To support these claims, the manufacturers cite a non-peer reviewed report from Bulgaria on their website [215]. In this report, 24 healthy Olympic level soccer players were administered increasing doses of CrM-Alk or CrM at one-month intervals (i.e., 0, 1.5, 4.5, and 6 g/day). The authors reported that CrM-Alk promoted less of an increase in urine creatinine than CrM despite changes being <0.2% different between groups at each time point; that urine pH increased by 0.65 in the CrM-Alk group, but only 0.1 in the CrM group (CrM-Alk 5.27 to 5.92; CrM 5.5 to 5.6); and, peak oxygen uptake increased in the CrM-Alk group (<30 mL/min over time or <1.0% for a trained individual). No differences in body weight were reported. These investigators concluded the CrM-Alk group “outperformed creatine monohydrate as a creatine product” despite not performing any statistical analysis to determine if these minimal differences were statistically significant. Thus, the report does not validate claims than CrM-Alk supplementation is a more bioavailable, efficacious, or safer form of creatine than CrM.

**Figure 13:**
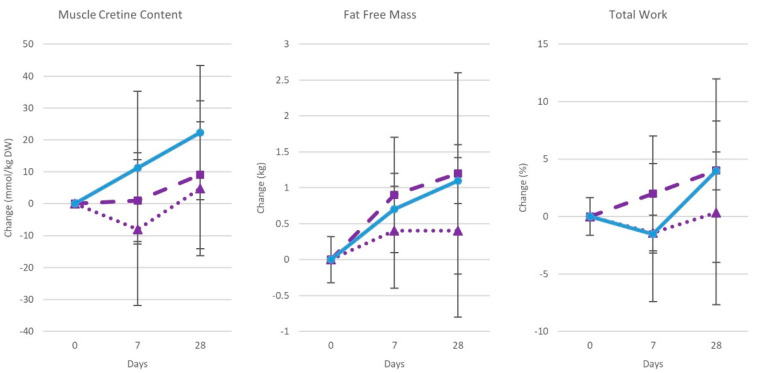
Changes in muscle creatine content, fat-free mass, and 30 s cycling sprint performance after 7 and 28 days of CrM-Alk supplementation at 1.5 g/day recommended doses (▲), CrM-Alk supplementation of 20 g/day for 7 days and 5 g/day for 21 days (◼), or CrM supplementation of 20 g/day for 7 days and 5 g/day for 21 days (●). Adapted from Jagim et al. [28].

Conversely, in a very well-controlled clinical trial, Jagim and colleagues [28] compared the effects of CrM-Alk supplementation at recommended and equivalent doses to CrM during 28 days of training in resistance-trained athletes with no recent history of creatine supplementation. In a double-blind manner, 36 resistance-trained participants were randomly assigned to ingest CrM (4 × 5 g/day for 7-days, 5 g/day for 21-days), CrM-Alk at recommended doses (1.5 g/day for 28-days), or CrM-Alk with equivalent doses to CrM (4 × 5 g/day for 7-days, 5 g/day for 21-days). Muscle biopsies, dual-energy x-ray absorptiometry (DXA) determined body composition, and performance measures were obtained after 0, 7, and 28 days of supplementation. Results revealed that neither recommended doses of CrM-Alk or loading and maintenance equivalent doses of CrM-Alk to CrM promoted greater changes in muscle creatine content, body composition, strength, or anaerobic capacity than CrM (see Figure 13). In fact, muscle creatine content was not significantly increased after 7 or 28 days of supplementation at recommended doses (−6.4  ±  37.8; 13.7  ±  42.2 %, respectively). There was some evidence that ingesting higher doses of CrM-Alk increased muscle creatine content after 28 days (6.2  ±  29.2; 27.3  ±  49.1%, respectively), but these values were less than observed with CrM (23.5  ±  49.0; 50.4  ±  44.8%, respectively). Thus, while high doses of CrM-Alk may increase muscle creatine content to some degree over time, there is no evidence that CrM-Alk is up to 10 times more bioavailable than CrM and/or recommended doses are efficacious. Additionally, was no evidence that CrM-Alk promoted greater training adaptations than those taking CrM or that participants taking CrM-Alk experienced fewer side effects than those taking CrM. Therefore, buffered creatine monohydrate is classified in some evidence to the support bioavailability, efficacy, and safety categories, but there is no evidence that buffered creatine is better.

Table 3 summarizes results of studies assessing creatine-containing compounds in which there is some evidence supporting bioavailability, efficacy, and/or safety. As described above, while some effects were observed compared to placebo ingestion and in some instances have comparable effects on performance as CrM, none of these forms have been shown to promote greater creatine retention in muscle than CrM.

## 10. No Evidence to Support Bioavailability, Efficacy, and Safety

### 10.1. Other Creatine Salts

While there is some bioavailability, efficacy, and safety data on CC and CPY, little to no data are available on several other creatine salts listed in Table 1. A patent from Negrisoli and Del Corona [31] disclosed several hydrosoluble organic salts of creatine including creatine maleate, creatine fumarate, creatine tartrate, and creatine malate. The inventors claimed that these creatine salts had solubilities of 10, 19, 3, 8.5, and 4.5 g/100 mL (%), respectively. Since salts are relatively weak bonds, it is likely that these creatine salts would increase creatine in the blood at equimolar doses that are generally about 1.3–1.6 times greater than CrM. However, there are no data indicating that these creatine salts increase blood creatine content, increase tissue creatine content, have any ergogenic value, are safe for long-term supplementation, or are more effective sources of creatine than CrM. A patent disclosing creatine ascorbate was also filed in the late 1990s [33]. The rationale was to provide a means of increasing creatine and ascorbic acid availability to improve exercise capacity while supporting the immune system [33]. However, we are not aware of any pharmacokinetic or exercise related studies to test this hypothesis and benefits would seemingly be similar and more effectively dosed by co-ingestion of CrM and vitamin C. Finally, there has been interest in tri-creatine orotate (CO) as a creatine salt (71% creatine by molecular weight) and a few raw material suppliers offer CO as a source of creatine to manufacturers [30,176,177]. Supplement companies who sell creatine orotate claim it provides creatine and orotic acid that is purported to aid in the production of carnosine in the muscle, and therefore improves muscle-buffering capacity. However, as of this writing, we are aware of no data showing bioavailability and/or efficacy of CO supplementation [216]. The European Food and Safety Authority has also expressed concerns about the potential cancerogenic effects of orotic acid [176,177]. Therefore, CO does not seem to be a good alternative for CrM in dietary supplements particularly when co-ingestion of effective doses of CrM and beta alanine would seeming be more effective. Based on this analysis, these creatine salts are classified in the no evidence to support bioavailability, efficacy, and safety category. Therefore, they cannot be considered more effective than CrM.

### 10.2. Creatine Serum

**Figure 14:**
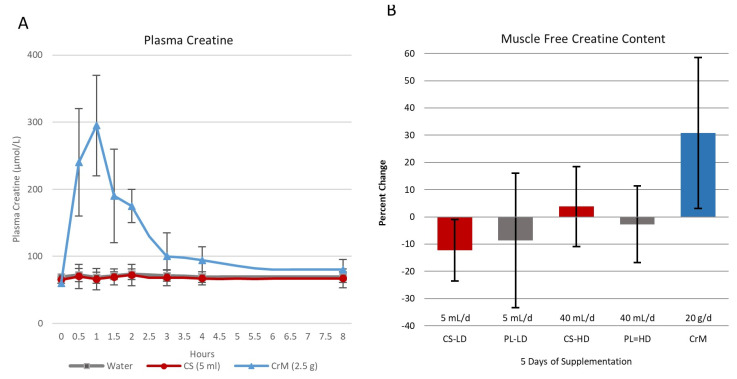
Changes in plasma creatine levels after oral ingestion of water, 5 mL of creatine serum (CS) purportedly providing 2.5 g of CrM, and 2.5 g of CrM in solution (**A**) [26] and 5 days of 5 mL of CS purportedly providing 2.5 g of creatine (CS-LD), 5 mL of a flavored placebo (PL-LD), 8 × 5 mL of CS (CS-HD) purportedly providing 20 g/day of creatine, 8 × 5 mL of flavored placebo (PL-HD), or 4 × 5 g/day of CrM (**B**) [26], Adapted from Harris et al. [38] and Kreider et al. [26].

As noted above, there has been interest in developing shelf-life stable liquid, gels, and/or beverages containing creatine. The theoretical rationale has been that these types of products may be more convenient to consume, absorbed faster into the blood, and/or promote a greater efficiency in transport of creatine to the muscle. One product that was heavily marked in the late 1990s and early 2000s is “creatine serum” (CS). This product claimed to deliver 2.5 g of creatine per 5 mL oral dose by providing a “creatine phosphate complex” that was designed to be absorbed via mucosal thereby bypassing the supposed degradation of creatine to creatinine through digestion [217]. Their rationale was based on general pharmacokinetic absorption studies indicating that drugs and/or nutrients are absorbed faster through the mucosal lining in the mouth. However, when researchers evaluated the creatine content of CS, they found that CS contained <10 mg of creatine and 69 mg of creatinine per 5 mL dose in multiple samples and lot numbers [38]. Additionally, they found that one 5 mL oral dose of CS purportedly providing 2.5 g of creatine had no effect on plasma creatine levels (same as water) whereas ingestion of 2.5 g of CrM increased plasma creatine levels to about 300 µmol/L after one hour of ingestion and declining in a classical manner throughout the next 8 h (see Figure 14A) [38]. No changes in creatinine levels were seen among participants ingesting CS, CrM or water. Consequently, this study shows that CS does not contain creatine and has no bioavailability in the blood [38]. To further assess the bioavailability of serum creatine, Kreider and colleagues [26] evaluated the effects of ingesting 5 mL of a flavored placebo; 5 mL of CS (purportedly providing 2.5 g of CrM); 8 × 5 mL doses of CS per day (purportedly providing 20 g/day of CrM); and 4 × 5 g doses of CrM (20 g/day) for 5 days on muscle creatine, phosphocreatine and content. Results revealed that CrM loading significantly increased total muscle creatine (+31%) and phosphocreatine (+16%) (see Figure 13). However, CS ingestion at recommended and equivalent doses had no effects on muscle free creatine, phosphocreatine, total creatine content, or ATP concentrations. Collectively, these studies show that CS is not a bioavailable source of creatine, and therefore can have no creatine-related efficacy. Therefore, it is classified in the no evidence to support category, and there is no evidence that CS outperforms CrM. Unfortunately, this product remains in the marketplace despite data showing it is a completely ineffective source of creatine.

### 10.3. Creatyl-L-Leucine

**Figure 15:**
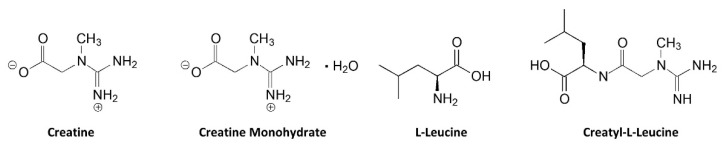
Comparison of creatine, creatine monohydrate, L-leucine, and creatyl-L-leucine chemical structures.

Creatyl-L-Leucine (CLL) has been marketed as “super creatine” [218]. As described in a patent [219], CLL is claimed to be “stable aqueous composition” of an “amide-protected, biologically-active form of creatine (creatyl-amide) molecule” that is “stable across a wide range pH’s and temperatures” and “can provide a wide range of physiological benefits including, for example, regeneration of ADP to ATP in muscle tissue, increasing the serum concentration of creatine, increasing muscle fiber size/cross-sectional area and lean body mass, activating satellite cells, enhancing memory and cognitive function, enhancing the functional capacity of a mammal having a neuromuscular disease, increasing muscular strength, endurance and/or power, enhancing cognitive function in infants with inborn errors of creatine metabolism, and/or alleviating the deleterious effects of sleep deprivation”. Analysis of the structure of CLL (see Figure 15) indicates that CLL does not contain a creatine molecule. Additionally, amide bonds are generally very strong, so pharmacokinetic data would need to show that CLL breaks down into creatine, increases creatine in the blood, and increases tissue creatine content to establish that CLL is a bioavailable source of creatine.

**Figure 16:**
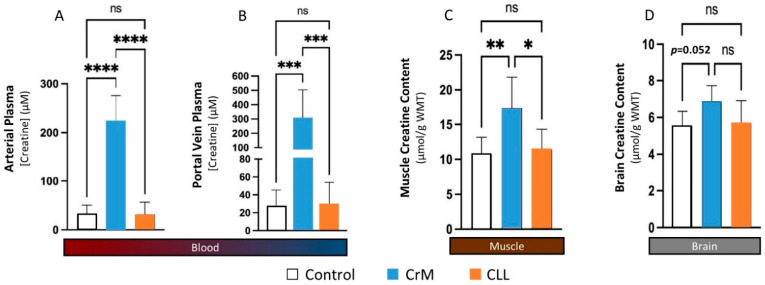
Blood, muscle, and brain creatine content in response to rats fed a control diet, 4.0 g/kg/day of creatine monohydrate (CrM), or 6.56 g/kg/day of creatyl-L-Leucine (CLL) for 7 days. (**A**) presents arterial plasma creatine concentration, (**B**) presents portal vein creatine concentration, (**C**) presents muscle creatine content, and (**D**) presents brain creatine content data for each group. Data are means ± standard deviations. **** = *p* < 0.0001, *** = *p* < 0.001, ** = *p* < 0.01, * *p* < 0.05, ns = not statistically significant between groups identified in brackets. Adapted from da Silva [221].

As of this writing, only two published articles have assessed the safety and/or efficacy of CLL. The first study was toxicology assessment of the administration of large doses of CLL in rats [220]. This study found that CLL did not cause mortality, toxic effects, or adverse effects in rats administered CLL for 90-days by oral gavage at doses of 1250, 2500, and 5000 mg/kg/day. More recently, da Silva [221] conducted an elegant study assessing the effects of feeding 24 rats either a control diet, a diet containing 4.0 g/kg/day of CrM, or a diet containing 6.56 g/kg/day of CLL for 7 days on arterial delivery of creatine, tissue uptake, and storage. According to the researchers, for a 70 kg individual, this would equate to a dose of 17.6 g/day of CrM and 28.9 g/day of CLL providing equimolar amounts of creatine if CLL based on the molecular weight of creatine if CLL degraded into creatine. As shown in Figure 16, rats fed CrM experienced significant increases in creatine concentrations in arterial plasma (+7-fold), portal vein plasma (+10-fold), muscle creatine content (+1.63-fold from control, and +1.53-fold from CLL) while tending to increase brain creatine content (*p* = 0.052) compared to controls. These changes were significantly greater than rats fed a control or CLL containing diet. Additionally, rats fed CLL did not increase blood, muscle, and brain creatine content above rats fed a control diet. The researcher concluded that provision of large doses of CLL to rats did not increase creatine bioaccumulation indicating that CLL is poorly absorbed by the intestine and is not a bioavailable source of creatine.

Several other studies have been recently conducted on CLL supplementation with human participants by experienced researchers at respected institutions with reports of results submitted in ongoing lawsuits [222,223,224,225,226]. These reports provide additional data showing CLL is not degraded into creatine upon oral ingestion [222,223,226], CLL does not increase blood creatine content [222,223,226], and CLL does not increase muscle [224,225] or brain creatine content [224] even when administered at doses much higher than found in marketed products containing CLL in humans [222,223,224,225,226]. Additionally, several of these studies reported that ingesting equivalent doses of CrM promoted significantly greater increases in blood [222,223,226] and tissue creatine content [225] than those ingesting CLL and CLL ingestion was no different than placebo controls [225,226]. Thus, available evidence indicates that CLL is not creatine, CLL is not a bioavailable source of creatine, and CLL is not “super creatine” compared to CrM. Therefore, CLL is listed in the no evidence category.

### 10.4. Creatinol-O-Phosphate

**Figure 17:**
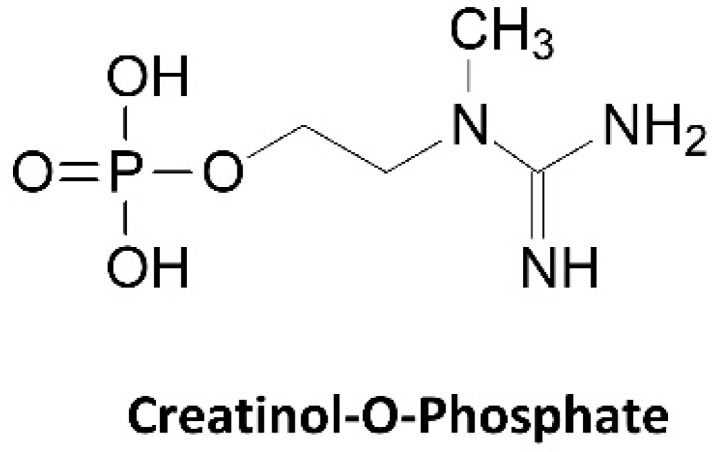
Chemical structure of Creatinol-O-Phosphate.

Figure 17 shows the chemical structure of creatinol-O-phosphate (COP). Creatinol in the form of COP is not creatine, nor was it intended to increase muscle creatine content. Rather, it was initially studied in the 1970s to intravenously deliver phosphate to improve myocardial function and reduce arrhythmias during ischemic conditions [227,228,229,230]. For example, Melloni and colleagues [228] investigated the effects of intravenous administration of 1020 mg, 2040 mg, and 3060 mg of COP compared to placebo on arterial blood pressure, heart rate and arrhythmias. The researchers found that COP administration increased blood phosphate levels as well as urinary excretion of phosphate and creatinine. Phosphate loading has been found to increase myocardial ejection fraction during exercise and maximal aerobic capacity [231,232] and is considered an ergogenic aid for endurance athletes [14]. While this is unrelated to creatine supplementation, the increase in urinary creatinine excretion led some to speculate that creatinol may act as a precursor of creatine and thereby serve as a source of creatine the body [228]. However, pharmacokinetic studies indicated that absorption of COP was complete when administered intramuscularly and distributed primarily to the kidney, liver, and heart and that COP could cross myocardial cell membranes [230]. One study from 1975 has been reported by others to show that intramuscular and intravenous administration of COP increased handgrip performance [233]. However, it is difficult to find details about this study. We are also not aware of any study that has evaluated whether oral COP has any effect on muscle creatine levels or exercise performance. Nevertheless, some companies have included COP as a source of creatine in dietary supplements and energy drink beverages. There is no evidence that oral COP ingestion has any effect on muscle creatine content or creatine-related metabolism. Claims that oral COP is a source of creatine and/or is more bioavailable than CrM are not supported.

Table 4 summarizes the results of studies that have evaluated sources of creatine that currently have no evidence supporting of bioavailability, efficacy, and/or safety. As can be seen, there are limited published data on these purported sources of creatine, and the available evidence indicates that they are not bioavailable sources of creatine.

## 11. Regulatory Status

### 11.1. United States

In the United States (US), congress enacted the Dietary Supplement Health and Education Act (DSHEA) of 1994 that placed dietary supplements in a special category of foods under the jurisdiction of the FDA. According to DSHEA, a dietary supplement is a product intended to supplement the diet, ingested orally, and contains a “dietary ingredient”. Dietary ingredients include vitamins, minerals, amino acids, herbs, botanicals, and other substances such as extracts, metabolites, or concentrates of those substances [14,234]. Dietary supplements can be delivered in powders, pills, capsules, hard and chewable tablets, soft gels, gummies, liquids, and even properly labeled energy bars that are intended for oral ingestion. However, they cannot include products promoted for sublingual, intranasal, transdermal, injected, or in any other route of administration [14]. DSHEA also established laws for FDA oversight over “new dietary ingredients” (NDI), which are ingredients that introduced to the marketplace after DSHEA was enacted [235]. A dietary supplement containing an NDI is deemed adulterated by the FDA, and therefore may not be lawfully distributed, unless (1) the NDI has “been present in the food supply as an article used for food in a form in which the food has not been chemically altered” or (2) there is a “history of use or other evidence of safety” that is submitted to the FDA for at least 75 days before selling the product (i.e., an “NDI Notification”). Dietary ingredients that were sold in the U.S. prior to October 15, 1994, were considered “grandfathered,” and therefore not NDIs subject to these requirements. This included CrM since it was introduced into the U.S. market in 1993 [236].

Since all of the other purported sources of creatine described above were introduced into the U.S. marketplace after 15 October 1994, they are considered NDI’s and manufacturers and distributors were expected to notify the FDA about these ingredients (See Section 413(d) of the Federal Food, Drug, and Cosmetic Act (the FD&C Act), 21 U.S.C. 350b(d)) [235] unless they meet the “present in the food supply” exemption noted above. An NDI Notification should include documentation of how the product containing the NDI is “reasonably expected to be safe” along with (1) the name of the new dietary ingredient (or Latin binomial name if it is an herb or botanical); and (2) “a description of the dietary supplement that contains the new dietary ingredient, including (a) the level of the new dietary ingredient in the product, (b) conditions of use of the product stated in the labeling, or if no conditions of use are stated, the ordinary conditions of use, and (c) a history of use or other evidence of safety establishing that the dietary ingredient, when used under the conditions recommended or suggested in the labeling of the dietary supplement, is reasonably expected to be safe” [14]. Once submitted, the FDA has 75 days to object to the notification. If the FDA does not respond within this timeframe, the NDI can be included in dietary supplements and legally sold in the U.S. market. However, it is important to understand that an NDI Notification only indicates that the FDA considers the NDI to be reasonably be considered as safe for human consumption. It does not affirm efficacy and/or validate any claims made about the NDI.

Since DSHEA and FDA regulations do not provide sufficient clarification on many issues, there has been a lot of confusion in the dietary supplement industry on what is an NDI, what manufacturing or other changes made to an ingredient cause it to be a “new” ingredient, when an NDI Notification is required, and what information it should contain. To provide clarification, the FDA released a “Draft Guidance for Industry” entitled “Dietary Supplements: New Dietary Ingredient Notifications and Related Issues” in July of 2011. However, that draft guidance prompted even more confusion and controversy, so the FDA released a revised draft guidance in 2016. While a guidance does not carry enforcement authority like a law or regulation, it provides the FDA’s perspective of how they interpret the laws and regulations related to NDI’s to help dietary supplement manufacturers know whether they are required to submit an NDI Notification to FDA, how to prepare NDI Notifications consistent with FDA review expectations, and how to improve the quality of submissions [235]. The 2016 Draft Guidance has also been criticized for a lack of clarity concerning what was considered a grandfathered ingredient and whether an NDI Notification was required if another manufacturer had already submitted an NDI Notification among other issues. This led to ingredients that should have been considered to be NDIs entering the marketplace with a notification, and several NDI Notifications being rejected by the FDA for lack of adequate safety data and/or other issues.

As an alternative to submitting an NDI Notification, some companies have pursued obtaining “Self-Affirmed” “Generally Recognized as Safe” (GRAS) status by conducting toxicology studies on animals and having scientific experts review the safety data and affirm the ingredient was reasonably expected to be safe. In this case, the company commissions studies to assess safety (e.g., toxicology studies in animals) and maintains internal documents to show safety if requested by the FDA. Once an ingredient is self-affirmed as GRAS, it can be introduced into the food supply, and is then not required to submit an NDI Notification to FDA to be included in dietary supplement under the “present in the food supply” exemption noted above. Companies can voluntarily submit their ingredient’s GRAS determination to FDA, however, there is no requirement to do so, and therefore a company’s GRAS self-affirmation can remain private. While lawful, the FDA has expressed some concern about this approach and discourages dietary supplement manufacturers from self-affirming GRAS to avoid submitting an NDI Notification.

After a dietary supplement is entered into the U.S. market, the FDA can restrict or ban its sale if it is deemed adulterated (e.g., unsafe). The FDA works with the Federal Trade Commission (FTC), who has jurisdiction over marketing claims that companies make about dietary supplements in advertising to regulate the dietary supplement industry. The FTC can act against companies for disseminating false, misleading, or unsubstantiated claims about dietary supplements. Marketing claims made for dietary supplements can also be challenged in litigation brought by consumers or competitors. This background provides the basis for understanding how various purported forms of creatine have entered the US market. The legal and regulatory status of CrM in dietary supplements is indisputable because it appeared on the US market in 1993 [236], and there was a large body of evidence showing it was reasonably expected to be safe. Since then, AlzChem Trostberg GmbH (the Germany manufacturer of CrM) voluntary submitted a GRAS application to the FDA that was not acted upon, meaning it could claim that CrM has FDA approved GRAS status [237,238]. Consequently, CrM is considered GRAS for inclusion as a dietary ingredient in dietary supplements, energy drinks, protein bars and powders, milkshakes, meal replacement powders and bars, meat replacement products, powdered drink mixes, and functional foods. As of this writing, CrM is the only form of creatine that is listed on the FDA’s inventory of GRAS notices [239] (see Table 5). As noted above, a company is not required to submit its GRAS self-affirmation to FDA, and therefore it may not be public information.

The legal status of other purported sources of creatine is less clear. According to the FDA’s NDI Notification database, since 1995, NDI Notifications for creatine pyruvate (1998), creatine ethyl ester (2003), creatine ethyl ester HCL (2004), tri-creatine orotate (2003), β-creatine (2010), creatine nitrate (2011 and 2017), and creatine acesulfame (2018) have been submitted. Several of these notifications were initially objected to by the FDA citing: (1) the form of creatine may not be a legal dietary ingredient as defined by the FD&C Act §201(ff); (2) inadequate safety information to conclude that the form of creatine is reasonably expected to be safe; and/or (3) a lack of information about the chemical identity of the creatine form [25]. However, creatine pyruvate at doses of 5–10 g/day and creatine nitrate at 750 mg per day have not been objected to by the FDA and can be sold as dietary ingredients. The AHPA NDIN database does not include notifications for creatine maleate, creatine fumarate, creatine tartrate, creatine ascorbate, creatine citrate, magnesium creatine chelate, creatine HCL, alkaline creatine (although it is a buffered form of CrM), creatine serum, CLL, or COP. Since some of these sources of creatine are ingredients in dietary supplements, any company selling these nutrients would have to have documentation that the ingredient was on the market in the US before 15 October 1994 or is present in the food supply in a form that is not chemically altered. While FDA GRAS notifications are published and are accessible to the public, self-affirmed GRAS files are not published, which makes them difficult to search and to validate its content. Based on a press release, we identified one self-affirmed GRAS affirmation for a creatine chelated with Mg (Creatine MagnaPower^®^).

### 11.2. International Regulation

Every country has independent laws and regulations governing dietary supplements. Some follow FDA guidance while others classify dietary supplements and drugs and have additional oversight, approval, and/or limitations about dosages [240]. Creatine monohydrate can be legally sold throughout the world although some countries limit the amount to of CrM that can be included per dose (e.g., no more than 3–5 g/serving). Some of the other forms of creatine marketed as ingredients for dietary supplements are not permitted to be included in dietary supplements in their country due to a lack of safety data. Therefore, except for CrM, one cannot assume that of the purported other sources of creatine described above can be legally sold as a dietary supplement in all countries. The following provides a brief overview in major markets that creatine is sold and regulatory oversight. Table 6 describes the responsible agencies and regulatory status of creatine containing dietary supplements in various countries. As can be seen, CrM remains the only source of creatine that is approved for sale in Australia, Canada, China, the European Union, Japan, and South Korea. Additionally, it is the only source of creatine that has approved health claims in the European Union, Canada, Japan, and South Korea.

### 11.3. Assessment and Guidance for Industry

The regulatory status of CrM is unequivocal in the global markets as a dietary or food supplement [25]. However, the scientific basis and regulatory status of other forms of creatine continues to be less clear. Since our last review in 2011, more data is available about several forms of creatine and several now have data providing some support as to efficacy and safety while others do not. No alternative form of creatine has shown superior bioavailability, efficacy or safety compared to CrM. Consequently, despite marketing hyperbole, CrM remains the gold standard to compare other forms of creatine with the strongest bioavailability, efficacy, and safety portfolios. Alternatives to CrM continue to be prevalent in the marketplace, including several that do not appear to meet regulatory requirements in several countries.

In the US, a major factor in determining whether an NDI notification is required to be submitted to the FDA is whether the NDI has been present in the food supply and/or has been chemically altered from its original form [26]. Any change in the chemical structure of an ingredient is assumed to alter the biological activity of the ingredient thereby requiring toxicology studies to establish that the NDI is reasonably expected to be safe as altered and that the biological behavior is comparable to the native ingredient. Most of the forms of creatine listed in Table 1 have been chemically altered in some way (e.g., covalently binding or complexing) to the creatine molecule. Some have clearly rearranged the creatine molecule. Therefore, they should have all been submitted as and NDI notification to the FDA prior to marketing. Yet, as described above, only nine of these newer forms are listed in the IND notification inventory. Of these, the FDA initially rejected some of the IND notification applications, yet the forms were sold for years before finally submitting an acceptable IND notification application or Self-Affirming GRAS. Even then, many have little to no data supporting bioavailability and/or efficacy despite making bold claims that the source is more bioavailable, effective, and/or a safer form of creatine than CrM.

The reason why alternate marketed forms of creatine are in the marketplace without pre-market IND notification is likely due to confusion over legal definitions of dietary supplements, natural health products, and/or food additives in different countries as well as what is meant by chemical alteration in a nutrient. Regardless, confusion of over laws regulating dietary supplements combined with inadequate enforcement by regulators has created an environment where there are often little consequences of non-compliance. For example, studies published in 2003 clearly showed that creatine serum was not a bioavailable source of creatine, yet it continues to be sold as a creatine-containing product. While alternative forms of creatine are unlikely to pose a health risk, they are typically more expensive than CrM. Additionally, misleading claims that lower doses of an alternate form of creatine are as effective as CrM may limit the benefits consumers may achieve from creatine supplementation. Given the health benefits of creatine, availability of ineffective sources of creatine or recommendations to take less creatine than needed to increase creatine stores in the muscle and/or brain can limit the benefits theses populations may derive from creatine supplementation [5]. Thus, we give the following recommendations as guidance to researchers and industry as they consider developing new dietary ingredients containing creatine.

(1) Only consider developing creatine supplements that contain a creatine molecule. Alteration of the chemical structure of creatine in any way is assumed to change the chemical activity and biological function and may negate any benefit of creatine supplementation. Additionally, binding creatine to other compounds may prevent creatine from being liberated in vivo, thereby making the form of creatine non-bioavailable or less bioavailable source of creatine.
(2) Companies who develop new forms of creatine should conduct toxicology studies in animals to establish that high dose ingestion is safe and conduct clinical trials in humans to validate safety. We then recommend obtaining FDA GRAS status or Self-Affirming GRAS status.
(3) Pharmacokinetic studies must be performed to show that the novel form of creatine is degraded into creatine and increases blood creatine levels to physiological levels necessary to promote creatine uptake into tissue (e.g., >200–500 µmol/L or 25–65 µg/L).
(4) Bioavailability studies should be conducted to show recommended doses increase muscle and/or brain creatine content.
(5) Placebo, double blind, and randomized clinical trials should be performed to substantiate that the form of creatine provides ergogenic benefit and does not cause any untoward side effects.
(6) Comparative effectiveness trials at recommended and equivalent doses must be performed to show a new form of creatine increases muscle and/or brain creatine content to a greater degree than CrM to substantiate those claims.
(7) Comparative effectiveness trials at recommended and equivalent doses must also be performed to determine if a new form of creatine is more effective and/or a safer alternative to CrM to substantiate those types of claims.
(8) Supplement companies should clearly declare the source and amount of creatine contained in their products so consumers can know if they are taking effective doses.
(9) Claims made about a form of creatine should be based on research conducted on that form of creatine at recommended doses, not untested hypotheses, speculation, assumptions, and/or marketing hyperbole. Such practices only undermine the scientific validity and consumer confidence about creatine supplementation.
(10) Pure CrM is the only source of creatine with strong evidence of bioavailability, efficacy, and safety and considered as GRAS by the FDA, approved for use in the EU and Australia, and evaluated for safety by Health Canada.
(11) Consumers should only consider taking supplements that contain sources of creatine that research has shown is bioavailable, effective, safe, and devoid of impurities.

## 12. Summary

CrM supplementation increases muscle phosphagen levels, improves repetitive high-intensity exercise performance, and promotes greater training adaptations [15]. No significant side effects other than weight gain have been reported from CrM supplementation despite widespread use throughout the world. Research on CrM has served as the basis to establish professional guidelines, recommendations, and establish regulation. CrM remains the only source of creatine that has substantial evidence of bioavailability, efficacy and safety and is considered GRAS by the U.S. FDA, is approved for use with accompanying health claims in the EU, has been extensively reviewed and approved by Health Canada, and is approved to be sold in major global markets. The bioavailability, efficacy, safety, and regulatory status of other purported sources of creatine are less clear, with only a few having some data supporting efficacy compared to placebo (see Table 7). However, there is no evidence that other “forms” of creatine are more bioavailable, effective, or safer forms of creatine compared to CrM. We recommend that companies interested in developing and marketing novel forms of creatine ensure the purported source contains the creatine molecule and conduct high-dose safety data in animals, pharmacokinetic studies to show the source of creatine increases blood and tissue concentrations of creatine, and comparative effectiveness studies to support structure and function claims. Additionally, the should company clearly list the amount of creatine contained in the supplement on supplement facts labels so consumers can make an informed decision about whether that purported source of creatine may deliver enough creatine to increase tissue creatine content by physiological levels needed to effect exercise and/or health.

## Figures and Tables

**Table 1:** Theoretical creatine content of purported sources of creatine-related compounds in the PubChem database based on molecular weight.

Compound	Molecular Formula	Molecular Weight (g/mol)	Theoretical Percent Creatine by MW ^†^	Difference from CrM (%)
Creatine (Creatine Anhydrous)	C_4_H_9_N_3_O_2_	131.13	100.0	13.8
Creatine Monohydrate	C_4_H_11_N_3_O_3_	149.15	87.9	0.0
Magnesium Creatine	C_4_H_9_MgN_3_O_2_	155.44	84.4	−4.0
Creatine Ethyl Ester	C_6_H_13_N_3_O_2_	159.19	82.4	−6.3
Methyl-Amino-Creatine	C_5_H_12_N_4_O_2_	160.17	81.9	−6.9
Creatine Hydrochloride	C_4_H_10_ClN_3_O	167.59	78.2	−11.0
Creatine Methyl Ester Hydrochloride	C_5_H_12_ClN_3_O_2_	181.62	72.2	−17.9
Creatine Nitrate	C_4_H_10_N_4_O_5_	194.15	67.5	−23.2
Creatinol-O-Phosphate	C_4_H_12_N_3_O_4_P	197.13	66.5	−24.3
Tri-Creatine Citrate	C_14_H_26_N_6_O_11_	585.50	67.2	−23.5
Phospho-Creatine	C_4_H_10_N_3_O_5_P	211.11	62.1	−29.3
Creatine Pyruvate	C_7_H_13_N_3_O_5_	219.20	59.8	−31.9
Creatine Beta-Alaninate	C_7_H_16_N_4_O_4_	220.23	59.5	−32.3
Creatine Lactate	C_7_H_15_N_3_O_5_	221.21	59.3	−32.6
Creatine Benzyl Ester	C_11_H_15_N_3_O_2_	221.26	59.3	−32.6
Di-Creatine Citrate	C_14_H_26_N_6_O_11_	454.39	57.7	−34.3
Creatine Sulfate	C_4_H_11_N_3_O_6_S	229.21	57.2	−34.9
Creatine Pyruvate Monohydrate	C_7_H_15_N_3_O_6_	237.21	55.3	−37.1
Di-Acetyl Creatine Ethyl Ester	C_10_H_17_N_3_O_4_	243.26	53.9	−38.7
Creatine Sulfate Monohydrate	C_4_H_13_N_3_O_7_S	247.23	53.0	−39.7
Creatine Ethyl Ester Pyruvate	C_9_H_17_N_3_O_5_	247.25	53.0	−39.7
Sodium Creatine Phosphate	C_4_H_8_N_3_Na_2_O_5_P	255.08	51.4	−41.5
Creatine Taurinate	C_6_H_16_N_4_O_5_S	256.28	51.2	−41.8
Creatine Pyroglutamate	C_9_H_16_N_4_O_5_	260.25	50.4	−42.7
Creatine Malate	C_8_H_15_N_3_O_7_	265.22	49.4	−43.8
Creatine Glutamate	C_9_H_16_N_4_O_6_	276.25	47.5	−46.0
Creatine Orotate	C_9_H_13_N_5_O_6_	287.23	45.7	−48.1
Creatine Carnitine	C_11_H_24_N_4_O_5_	292.33	44.9	−49.0
Creatine Ethyl Ester Malate	C_10_H_19_N_3_O_7_	293.27	44.7	−49.1
5-Hydroxytryptamine Creatine	C_14_H_21_N_5_O_3_	307.35	42.7	−51.5
Creatine Trinitrate	C_4_H_12_N_6_O_11_	320.17	41.0	−53.4
Creatine α-ketoglutarate	C_11_H_20_N_4_O_7_	320.30	40.9	−53.4
Creatine Citrate	C_10_H_17_N_3_O_9_	323.26	40.6	−53.9
D-Gluconic Acid Creatine Salt	C_10_H_21_N_3_O_9_	327.29	40.1	−54.4
Creatine Monohydrate Dextrose	C_10_H_23_N_3_O_9_	329.30	39.8	−54.7
Creatine Hydroxycitrate	C_10_H_17_N_3_O_10_	339.26	38.7	−56.0
Disodium Creatine Phosphate Tetrahydrate	C_4_H_18_N_3_Na_2_O_10_P	345.15	76.0	−13.6
Creatine Phosphate Lactate	C_13_H_22_N_3_O_15_P	491.30	26.7	−69.6
Creatine-CoA	C_25_H_43_N_10_O_17_P_3_S	880.70	14.9	−83.1

Adapted from Jaeger et al. [25]. MW represents molecular weight. ^†^ represents theoretical creatine content based on the MW of creatine assuming a full molecule of creatine is in the compound, and it is liberated as creatine from the compound. Many sources have not been studied to verify ingestion increases blood levels of creatine or tissue creatine content. Therefore, listing a compound in this table does not validate if the marketed source contains creatine, is bioavailable as creatine, or is an effective source of creatine. Not listed on PubChem as a creatine-containing compound: creatine maleate, creatine fumarate, creatylglycine ethyl ester fumarate, polyethylene glycosylated creatine, polyethylene glycosylated creatine HCL, Creatine Serum, creatyl-L-leucine.

**Table 2:** Example of studies showing bioavailability, efficacy, and safety of CrM supplementation.

Reference	Participants	Design	Duration	Dosing Protocol	Findings	Side Effects
Short-term Studies (<14 Days)						
Greenhaff et al. [71]	8 healthy males	SB	5 days	4 × 5 g CrM	CrM ↑ TCr by 25% and PCr resynthesis following electrically evoked isometric contractions.	None reported
Balsom et al. [161]	7 males	SB	6 days	4 × 5 g CrM	↑ in total muscle total creatine (18%), weight (1.1 kg), and 5 × 6 s cycling sprint performance and PCr recovery	None reported
Green et al. [20]	24 healthy men	RDBP	5 days	4 × 5 g CrM followed by 93 g CHO or CHO	Ingesting CrM with CHO ↑ muscle TCr and glycogen	None reported
Vandenberghe et al. [162]	9 healthy non-vegetarian males	RDBPC	5 days with 5 week washout	25 g/day CrM or PLA	CrM ↑ muscle PCr by 11% and 16% after 2 and 5 days. PCr resynthesis rate was not affected.	None reported
Bellinger et al. [163]	20 endurance cyclists	RDBP	7 days	20 g/day CrM or PLA	CrM ↑ muscle creatine content by 30% and decreased TAN contribution to sprint	None reported
Francaux et al. [164]	14 physically active males	RDBP	14 days	3 × 7 g of CrM or PLA	CrM ↑ MRS PCr by ~20% and PCr repletion by 15% and 10% during 40% and 70% MVCs.	None reported
Preen et al. [116]	14 physically active men	RDBP	5 days	20 g/day CrM or PLA	CrM increase TCr stores and work during 80-min of repeated cycling sprint exercise.	None reported
Burke et al. [165]	20 male resistance-trained athletes (18–32 years)	RDBP	5 days	4 × 5 g CrM, 4 × 5 g CrM + 25 g Sucrose, or 4 × 5 g CrM + 25 g Sucrose + 250 mg α-LA or PLA	CrM ↑ body weight (2.1 kg) with no differences among groups, TCr was ↑ more in the CrM + sucrose + α-LA group.	None reported
Longer-Term Studies (>14 days)						
Vandenberghe et al. [47]	19 young female volunteers	RDBP	10 weeks phase I (*n* = 19); 10 weeks phase II (*n* = 13)	4 × 5 g CrM for 4 days, 5 g/day thereafter or PLA	CrM ↑ muscle PCr, strength, and exercise capacity	None reported
Kreider et al. [55]	25 American college football players during offseason resistance and agility training	RDBP	28 days	CrM 15.75 g/day with glucose or glucose PLA	↑ FFM, ↑ strength, ↑ muscular endurance, ↑ 6 × 6-s cycling sprint performance with 30-s rest	None reported
Volek et al. [113]	19 healthy resistance-trained males	RDBP	12 weeks	CrM 5 × 5 g for 7 days, 5 g/day for 11 weeks or PLA	↑ FFM, strength, and muscle morphology	No differences
Kreider et al. [166]	51 American college football players during offseason resistance and agility training and spring football	RDBP	12 weeks	20 g/day and 25 g/day of CrM with CHO and PRO; CHO only; or CHO + PRO only	CrM groups ↑ FFM, ↑ strength, ↑ muscular endurance. No changes in blood chemistry panels.	CrM groups had less GI complaints than those ingesting CHO and CHO + PRO.
Tarnopolsky et al. [167]	23 young healthy but untrained males	RDBP	8 weeks	10 g/day CrM with 75 g CHO or PLA	CrM with CHO promoted greater ↑ in body mass and FFM during training.	None reported
Willoughby et al. [168]	22 untrained males during resistance-training	RDBP	12 weeks	CrM 6 g/day or PLA	CrM promoted > increases in body mass, FFM, thigh volume, muscle strength, myofibrillar protein content, and myosin heavy chain mRNA expression for Type I, IIa, and IIx fibers	None reported
Burke et al. [108]	18 vegan and 24 non-vegan (20 men, 22 female)	RDBP	56 days	0.25 g/kg FFM/d of CrM for 7 days, 0.0625 g/kg FFM/d for 49 days or PLA	TCr content was lower in vegans. CrM ↑ PCr, TCr, and gains in bench press strength, isotonic work, Type II fiber area, and FFM during resistance training.	None reported
Lyoo et al. [73]	15 males (23–35 years)	RDBP	56 days	2 × 0.15 g/kg CrM for 7 days, 2 × 0.015 g/kg CrM for 49 days or PLA	CrM ↑ brain PCr (3.4%), Pi (9.8%), and Cr (8.1%) while decreasing β-nucleoside triphosphate (NTP) by 7.8%.	None reported
Newman et al. [169]	17 healthy active but untrained men	RDBP	33 days	4 × 5 g CrM + 3.75 glucose for 5-days, 3 g CrM + 3 g glucose thereafter or PLA	CrM ↑ muscle TCr after loading and maintenance doses. CrM had no effects on muscle glycogen, glucose tolerance or insulin sensitivity.	None reported
Tarnopolsky et al. [170]	Moderately active younger (13 men, 14 women; 19 resistance-trained men; Older resistance-trained men (15) and women (15)	RDBP	5 days; 8 weeks; 14 weeks	4 × 5 g CrM for 5 days; 10 g/day CrM with 75 g dextrose for 8 weeks during training; 5 g/day CrM + 2 g/day dextrose for 14 weeks during training or PLA	CrM ↑ muscle TCr in each study compared to placebo. CrM nor training influenced creatine transporter protein content. Citrate synthase was increased in older participants.	None reported
Willoughby et al. [171]	22 untrained males during resistance-training	RDBP	12 weeks	6 g/day CrM or PLA	CrM promoted > ↑ in muscle CK, myogenin, and MRF-4.	None reported

R = randomized; DB = double-blind; *p* = placebo; SB = single blind; C = crossover, CrM = creatine monohydrate, PCr = phosphocreatine; TCr = total creatine; Pi = inorganic phosphate; CHO = carbohydrate; PRO = protein; FFM = Fat-Free Mass, TAN = total adenine nucleotide pool; MVC = maximal voluntary contractions; α-LA = alpha lipoic acid; MRS = magnetic resonance spectroscopy; GI = gastrointestinal.

**Table 3:** Creatine containing compounds other than CrM with some evidence supporting bioavailability, efficacy, and/or safety.

Reference	Participants	Design	Duration	Dosing Protocol	Findings	Side Effects
Creatine Salts						
Jäger et al. [63]	3 females and 3 males	RDBPC	1 oral dose with 7 day washout	5 g CrM 6.7 g CC 7.3 g CPY	Creatine peak AUC was higher with CPY with no differences in absorption kinetics	None reported
Smith et al. [180]	15 recreationally active women (22.3 ± 0.6 yrs)	RDBP	5 days	20 g/day of CC	CC loading delayed the onset of neuromuscular fatigue during cycle ergometry.	None reported
Jäger et al. [174]	49 healthy males (26.5 ± 4 yrs)	RDBP	28 days	5 g/day of CC, CPY, or PLA	CPY and CC ↑ intermittent handgrip exercise of maximal intensity. Some evidence CPY might benefit endurance exercise.	None reported
Graef and coworkers [181]	43 recreationally active men (22.6 ± 5 yrs)	RDBP	5 days/week for 6-weeks	2 × 5 g/day of PLA or CC on training days	CC increases ventilatory anaerobic threshold (PLA 10%, CC 16%). No differences in time to exhaustion or total work.	None reported
Smith et al. [172]	55 active men (27) and women (28)	RDBP	5 days	4 × 5 g/day of CC or PLA	CC did not positively or negatively affect maximal aerobic capacity, critical velocity, time to exhaustion, or body mass.	None reported
Fukuda et al. [182]	50 recreationally active men (24) and women (26) 22 ± 3 yrs	RDBP	5 days	4 × 5 g/day of CC or PLA	CC loading ↑ anaerobic running capacity (+23%) with no effect in PLA group in men but not women.	None reported
Stone et al. [83]	42 American football players	RDBP	5 weeks	0.22 g/kg/day of PLA, CrM, caPYR, or CrM + caPYR	CrM and CrM + caPYR ↑ strength, FFM, and power output. No difference from PLA or caPYR alone.	GI issues with caPYR. None reported with CrM
Van Schuylenbergh et al. [175]	14 well-trained male endurance athletes (4 cyclists, 10 triathletes)	RDBP	7 days	2 × 3.5 g of CPY with 8 g CHO or PLA	CYP had no effects on 1-h time trial steady-state power output, interval sprints, total work lactate, or heart rate.	None reported
Nuuttilla et al. [187]	Olympic canoeists	RDBP	7 days	7.5 g/day of CPY or PLA	CPY improved paddle rate and lowered blood lactate suggesting an improvement in aerobic exercise efficiency.	None reported
***Magnesium Creatine Chelate***						
Brilla et al. [189]	35 recreationally active men	RDBP	14 days	800 mg/day magnesium (Mg) and 5 g/day Cr as Mg oxide plus Cr or MgCr-C	Body mass and power ↑ in both Cr groups while intracellular and extracellular water and peak torque only increased in the MgCr-C group	None reported
Selsby et al. [190]	31 resistance-trained men	RDBP	10 days	2.5 g/day of PLA, Cr or Mg-Cr	Both Cr groups improved bench press total work compared to PLA. No differences between groups.	None reported
Zajac et al. [191]	20 elite soccer players	RDBP	16 weeks	5.5 g/day of r MgCr-C or PLA	MgCr-C ↑ 35 m repeated sprint performance, total time, average power, and peak power with no changes in PLA group.	MgCr-C ↑ serum creatinine compared to PLA
Creatine Ethyl Ester						
Spillane et al. [27]	30 healthy males (20.4 ± 1.7 yrs)	RDBP	47 days	0.30 g/kg FFM for 5-days, 0.075 g/kg FFM for 42 days of PLA, CrM, or CEE	CEE ↑ in muscle TCr after 27-days compared to PLA. However, CrM observed significantly greater ↑ in TCr compared to PLA and CEE. CEE did not promote > training adaptations.	CEE ↑ serum creatinine twofold > than PLA and CrM. None reported with CrM.
Arazi et al. [197]	16 resistance trained males	RDBP	42 days	4 × 5 g/day of PLA or CEE for 5 days, 5 g/day for 37 days	CEE during resistance-training ↑ body weight and leg press strength while percent body fat ↓ with some evidence of an ↑ in testosterone and growth hormone.	None reported.
Creatine HCl						
de França et al. [65]	40 healthy males and females	RDBP	28 days	5 g/day PLA, 1.5 g/day of Cr-HCl, 5 g/day of Cr-HCl, or 5 g/day CrM	Reported some effects on skinfold determined fat mass and FFM and leg press strength but gains in CrM were greater than Cr-HCl	None reported.
Yoshioka et al. [201]	11 healthy elite Brazilian gymnasts	RDBP	30 days	5 g/dayay of CrM or 1.5 g/dayay of Cr-HCL with 3.5 g/dayay of resistant starch	Skinfold caliper determined FFM, strength, and BIA determined total body water was increased to a greater degree in the CrM group (CrM + 1.81 L vs. Cr-HCL +0.24 L).	None reported.
Tayebi et al. [202]	36 resistance trained men	RDBP	7 days	20 g/day CrM, 3 g/day CrM, 3 g/day Cr-HCL, or PLA	3 g/day of Cr-HCl did not promote greater gains in performance or hormonal responses than 3 or 20 g/day of CrM.	None reported.
Creatine Nitrate						
Ostojic et al. [209]	10 healthy men	RDBPC	1 oral dose	3 g CrN + 3 g CNN, 3 g CrN, 3 g CrM	CrN + CNN ingestion promoted a greater increase in serum creatine AUC levels (183.7 ± 15.5, 163.8 ± 12.9, and 118.6 ± 12.9 µmol/L, respectively).	None reported.
Ostojic et al. [209]	10 healthy men	RDBPC	5 days	3 g/day CrN + 3 g/day CNN, 3 g/day CrN, 3 g/day CrM	MRS determined muscle creatine content increased to a greater degree with CrN + CNN (9.6%, 8.0%, 2.1%, respectively)	Irregular bowel movement (1 CrN and CrN + CNN), Excessive sleepiness (1 CrN), Seldom stomach bloating (1 CrM). CrN + CNN decrease eGFR determined kidney function.
Galvan et al. [29]	13 males	RDBPC	1 oral dose with 7 day washout	1.5 g CrN (CrN-Low), 3 g CrN (CrN-High), 5 g CrM or a placebo	CrM ↑ plasma Cr AUC to a greater degree than PLA, CrN-Low, and CrN-High while plasma nitrate ↑ in CrN treatments.	None reported.
Galvan et al. [29]	48 active males	RDBP	28 days	4 × 5 g PLA, 4 × 1.5 g/day of CrN (CrN-Low), 4 × 3 g/day CrN (CrN-High), 4 × 3 g/day CrM for 7 days and 1 dose/d for 21 days	Creatine loading (12 g/day of CrM and CrN) ↑ muscle TCr in the CrM (7.1 mmol/kg DW) and CrN-High (4.6 mmol/kg DW) groups. CrM maintained ↑ muscle TCr. CrN-Low had no effects on TCr compared to PLA after 7 and 28 days. 3 g/day of CrN was not sufficient to maintain elevated muscle TCr after 28 days.	None reported.
Dalton et al. [36]	28 participants (18 men, 10 women)	RDBPC	6 days	3 g/day of PLA. 3 g/day CrN, 6 g/day CrN.	Up to 6 g of CrN for 6-days does not negatively affect resting hemodynamics, response to a postural challenge, the ability to perform high-intensity exercise, or clinical chemistry profiles.	None reported.
Joy et al. [212]	58 young males and females (24.3 ± 4 yrs)	R	28 days	Control group, 1 g/day CrN, 2 g/day CrN with other nutrients	1–2 g/day of CrN supplementation during training had no adverse effects on clinical blood chemistries compared to a non-supplemented group.	None reported.
Buffered Creatine						
Jagim et al. [28]	36 resistance trained males	RDBP	28 days	CrM (4 × 5 g/day for 7 days, 5 g/day for 21 days); CrM-Alk at recommended doses (1.5 g/day for 28 days); or CrM-Alk with equivalent doses to CrM (4 × 5 g/day for 7 days, 5 g/day for 21 days).	Neither recommended doses nor loading and maintenance equivalent doses of CrM-Alk promoted greater changes in muscle TCr, body composition, strength, or anaerobic capacity compared to CrM. Recommended doses did not ↑ TCr.	None reported.

R = randomized; DB = double-blind; *p* = placebo; SB = single blind; C = crossover; yrs = years; PLA = placebo, CHO = carbohydrate; PRO = protein; Cr = creatine; CrM = creatine monohydrate; CC = creatine citrate; CPY = creatine pyruvate; caPYR = calcium pyruvate, MgCr-C = magnesium creatine chelate, CEE = creatine ethyl ester; Cr-HCl = creatine hydrochloride; CrN = creatine nitrate; CNN = creatinine; CrM = Alk = buffered creatine; PCr = phosphocreatine; TCr = total creatine; AUC = area under the curve; FFM = Fat-Free Mass; BIA = bioelectrical impedance; GI = gastrointestinal; MRS = magnetic resonance spectroscopy; DW = dry weight.

**Table 4:** Creatine containing compounds that have no evidence of bioavailability, efficacy, and/or safety.

Reference	Participants	Design	Duration	Dosing Protocol	Findings	Side Effects
Creatine Serum						
Harris et al. [38]	6 males	R	1 oral dose with 7 day washout	Water Control, 5 mL CS (purportedly delivering 2.5 g CrM), 2.5 g CrM	CrM ↑ plasma Cr while CS had no effects and was similar to water. Analytic chemistry analysis showed < 10 mg of creatine and 90 mg creatinine in CS sample.	None reported.
Kreider et al. [27]	40 males (18–30 years)	RDBP	5 days	5 mL PLA, 5 mL of CS (recommended dose purportedly providing 2.5 g of CrM); 8 × 5 mL/day CS (purportedly providing 20 g/day of CrM); and 4 × 5 g doses of CrM (20 g/day)	CrM increased muscle creatine stores. Consumption of CS at recommended and 8× recommended levels had no effect.	None reported.
***Creatyl-L-Leucine***						
Reddeman et al. [189]	Rats	Open Label	90 days	Repeated-dose oral gavage toxicity study at doses of 1250, 2500, and 5000 mg/kg body weight per day.	There was no genotoxic activity observed in an in vivo mammalian micronucleus test at concentrations up to the limit dose of 2000 mg/kg body weight per day. The no observed adverse effect level from the 90-day study was determined to be 5000 mg/kg body weight per day, which was the highest dose tested for male and female rats.	None reported.
da Silva [221]	24 rats	R	7 days	Control diet, a diet containing 4.0 g/kg/day CrM, or a diet containing 6.56 g/kg/day CLL	CrM ↑ [creatine] in arterial plasma (+7-fold), portal vein plasma (+10-fold), muscle TCr (+1.63-fold from control, and +1.53-fold from CLL) while tending to increase brain creatine content compared to controls. CLL did not increase blood, muscle, and brain creatine content above rats fed a control diet with values lower than CrM.	None reported.
Creatinol-O-Phosphate						
Nicaise et al. [233]	-	-	-	Intramuscular and intravenous injection	COP↑ handgrip performance.	None reported.

R = randomized, *p* = placebo, DB = double-blind, SB = single blind; C = crossover, PLA = placebo, Cr = creatine; TCr = total creatine; CrM = creatine monohydrate, CS = creatine serum; CLL = creatyl-L-leucine; COP = creatinol-O-phosphate; ↑ = increase.

**Table 5:** Regulatory status of nutrients marketed as creatine supplements in the United States of America.

FDA Generally Recognized As Safe (GRAS)				
Purported Creatine Source Submitted	FDA Report Number	Submission Year	Intended Dosage	FDA Response
Creatine Monohydrate (Creapure^®^)	GRN 931	2020	1 g creatine (1.12 g creatine monohydrate) as an ingredient in “energy” drinks, protein bars and powders, milk shakes, meal replacement powders and bars, meat analogs, and powdered drink mixes (excluding infant formula.	FDA has no questions at this time.
Self-Affirmed Generally Recognized As Safe (GRAS)				
Purported Creatine Source Submitted	FDA Report Number	Submission Year	Intended Dosage	FDA Response
Creatine chelated with Mg (Creatine MagnaPower^®^)		2013		
New Dietary Ingredient Notifications (NDIN) *				
Purported Creatine Source Submitted	FDA Report Number	Submission Year	Intended Dosage	FDA Response
Creatine Pyruvate	RPT28	1998	5–10 g/day in 2 equal doses	Filed by FDA without substantive comments.
Creatine Ethylesters [Brand: Cre-Ester™]	RPT154	2002	Maximum daily dose of 30 g	Objected by the FDA.
Creatine Ethylesters [Brand: Cre-Ester™]	RPT190	2002	0.5–5.0 g/day	Objected by the FDA.
Tricreatine Orotate	RTP201	2003	1–2 g 3 ×/day (3–6 g/day)	Objected by the FDA.
Creatine ethyl ester HCL [Brand: CE2™]	RTP249	2004	500 mg–5 g/day	Objected by the FDA.
Creatine from creatine ethyl ester HCL [Brand: CE2™]	RTP264	2004	500 mg–3 g/day	Objected by the FDA.
Beta Creatine	RPT660	2010	4.5–7.5 g/day creatine 3–6 g/day beta-alanine	Objected by the FDA.
Creatine Nitrate	RTP696	2011	1.5 g serving, maximum dose 3 g/day	Objected by the FDA.
Creatine Nitrate	RPT993	2017	750 mg per day	Acknowledged with no objections by FDA.
Creatine acesulfame	RPT1064	2018	10 g per day	Objected by the FDA.

FDA = Food and Drug Administration. * Data retrieved from AHPA NDI Database http://ndi.ahpa.org/ (accessed on 1 January 2022).

**Table 6:** Regulatory status of nutrients marketed as creatine supplements primary international markets.

Country	Responsible Agency	Primary Regulations/Statutes	Regulatory Status of Creatine
Australia	Department of Health, Therapeutic Goods Administration (TGA).	Dietary supplements are considered complimentary medicines and regulated under the Therapeutic Goods Act (TGA) of 1989 [241] and 1990 TGA regulations [198]. Medicinal products are categorized as lower risk medicines that can be listed on the Australian Register of Therapeutic Goods (ARTG) [198] while higher risks medicines must be registered with the ARTG [241].	As of this writing, of the 90,988 products listed in the ARTG database, only 25 products contain creatine. Of these, CrM is the only source of creatine listed as an ingredient.
Canada	Health Canada [242]	Natural and Non-prescription Health Products Directorate (NNHPD) of Health Canada [242]. The NNHPD maintains a compendium of articles that reviews the safety and efficacy of licensed NHP’s [243]. CrM was assigned a monograph by the NNHPD that overviews research on CrM to substantiate safety and efficacy. Only products containing CrM can benefit from an abbreviated licensing process by referencing the monograph. Applicants using all other creatine forms are required to submit their own evidence of safety and efficacy for review as part of the pre-market licensing process.	As of this writing, 20 compounds purported to contain creatine are included in the NHP Ingredient Database [244] including creatine, creatine-alpha-ketoglutarate, creatine ethyl ester, creatine ethyl ester HCl, creatine gluconate, creatine HCl, creatine hydroxycitrate, creatine monohydrate, creatine nitrate, creatine orotate, creatine phosphate, creatine pyroglutamate, creatine pyruvate, creatine taurinate, dicreatine malate, disodium creatine phosphate, magnesium creatine chelate, polyethylene glycosylated creatine, polyethylene glycosylated creatine HCl, and tricreatine citrate. Creatinol-O-phosphate is listed as a medicinal product in the NHP database [244].
China	New Food Safety Law of the People’s Republic of China and the Administrative Permission Law of the People’s Republic of China, CFDA [198].	Nutritional supplements in China must be orally ingested, have at least one of 22 preventive functions as recognized by the Ministry of Health, and cannot be a curative drug [198]. Imported supplements must be approved by the National Medical Product Administration while foods are supervised by the State Administration for Market Regulation) [198].	Importers of dietary supplements and foods containing creatine must submit notification materials for review and approval before being allowed to be sold in China. Since CrM and other forms of creatine are produced in China, they would seemingly be legal to consume. However, it is unclear which forms of creatine are allowed to be imported into China.
European Union (EU)	European Commission Directive on Food Supplements [245,246,247]	The European Food Safety Authority (EFSA) evaluates scientific health claims. Creatine is considered a substance that may be added for specific nutritional purposes in foods for particular nutritional uses (FPNU) [246].	In 2004, EFSA indicated that the use of creatine in foods for nutritional use was not a matter of concern provided that the source had high purity (99.95%), did not contain impurities, and that dose of up to 3 g/day of supplemental creatine which is similar to the normal daily turnover rate of creatine was unlikely to pose any risk [248]. EFSA substantiated scientific health claims of CrM include: (1) CrM increases physical performance during short-term, high intensity, repeated exercise bouts, endurance capacity, and endurance performance [249], (2) CrM increases attention and improves memory [250], and, (3) CrM (at least 3 g/day) in combination with resistance training and improved muscle strength. All studies cited were performance on pure CrM so the regulatory status of other “forms” of creatine in the EU are less clear.
Japan	Ministry of Health, Labor and Welfare (MHLW)	Dietary substances in Japan are legally classified as food, food additives or “non-drug” (food). The Consumer Affairs Agency started “Foods with Function Claims” which reviews and approves health claims related to dietary supplements.	CrM is considered a “non-drug” [251] that is allowed to be sold as a food ingredient and additive under the Food Sanitation Law [252]. Health claims of CrM for muscle maintenance with exercise was accepted in 2019. Thus, CrM can be imported, distributed, and produced in Japan. CEE has been included on the “non-drug” list. In order for other forms to be imported, distributed, and/or produced in Japan, safety data and similarity of the proposed form to CrM must be submitted and approved by the MHLW [253]. In addition to CrM, creatine citrate and creatine pyruvate have been approved to be imported into Japan. It is unclear whether other forms of creatine can be imported into Japan and sold as dietary supplements.
South Korea	Ministry of Food and Drug Safety (MFDS) [254].	Similar to the U.S., new dietary ingredients must have sufficient safety data including toxicology studies in animals and supporting safety and efficacy data from human clinical trials to support efficacy at the recommended daily doses marketed.	An application to register CrM as a dietary supplement was filed in 2005 and approved by the MFDA for use as a dietary supplement 2008 with an accompanying health claim [255]. Given these requirements, forms of creatine reviewed above that have bioavailability data at recommended doses substantiating efficacy and safety seemingly be eligible for approval while those that do not have that data would likely experience more difficulty obtaining approval to sell their form of creatine in South Korea.

CrM = creatine monohydrate.

**Table 7:** Categorization of purported sources of creatine based on bioavailability, efficacy, and safety.

Strong Evidence	Some Evidence	No Evidence
Creatine Monohydrate	Creatine Citrate	5-Hydroxytryptamine Creatine
	Creatine Pyruvate	Creatine Benzyl Ester
	Magnesium Creatine Chelate	Creatine Beta-Alaninate
	Creatine Ethyl Ester	Creatine Carnitine
	Creatine HCl	Creatine Ethyl Ester Malate
	Creatine Nitrate	Creatine Ethyl Ester Pyruvate
	Buffered Creatine Monohydrate	Creatine Fumarate
		Creatine Gluconate
		Creatine Glutamate
		Creatine Hydroxycitrate
		Creatine Lactate
		Creatine Malate
		Creatine Maleate
		Creatine Methyl Ester HCL
		Creatine Monohydrate Dextrose
		Creatine Orotate
		Creatine Phosphate Lactate
		Creatine Pyroglutamate
		Creatine Pyruvate Monohydrate
		Creatine Serum
		Creatine Sulfate Monohydrate
		Creatine Taurinate
		Creatine Trinitrate
		Creatine α-ketoglutarate
		Creatine-CoA
		Creatinol-0-Phosphate
		Creatyl-L-Leucine
		Di-Acetyl Creatine Ethyl Ester
		Disodium Creatine Phosphate
		Methyl-Amino-Creatine
		Phospho-Creatine
		Polyethylene Glycosylated Creatine

## Data Availability

Not applicable.
